# Longitudinal Multi-Batch Characterisation of Beer Wort Fermentation Foam Using Image-Based Semantic Segmentation

**DOI:** 10.3390/foods15193366

**Published:** 2026-09-22

**Authors:** Anca Sipos, Otto Ketney, Mariana-Liliana Păcală, Romulus Iagarau

**Affiliations:** Department of Agricultural Science and Food Engineering, “Lucian Blaga” University of Sibiu, Dr. I. Rațiu Street, No. 7–9, 550012 Sibiu, Romaniamariana.pacala@ulbsibiu.ro (M.-L.P.); romulus.iagaru@ulbsibiu.ro (R.I.)

**Keywords:** alcoholic fermentation, foam, computer vision, deep learning, process monitoring, food industry

## Abstract

Image-derived descriptors of fermentation foam may complement conventional brewing-process measurements, but they require evaluation at the level of independent fermentation batches. We derived image-based estimates of foam height and foam-free exposed area from hourly images of 12 beer wort fermentations conducted at initial extract levels of 12, 15 and 18 °P, and analysed 1147 hourly observations together with synchronised pH, temperature and dissolved-oxygen records. Foam was segmented with convolutional encoder–decoder networks trained separately for each fermentation, using 223 annotated frames overall (19.4% of the 1147 acquired frames; 14–24 frames per batch), all acquired within the first 72 h. Because hourly frames were nested within fermentation batches, observation-level correlations were treated as exploratory. In a batch-aware sensitivity analysis based on 70 batch-by-window means, linear mixed-effects models with batch as a random intercept identified an effect of operational time window on foam height (F(5, 53.10) = 72.17; *p* < 0.001) and on foam-free exposed area (F(5, 62.00) = 80.57; *p* < 0.001), whereas an effect of initial extract level was not detected (*p* = 0.520 and *p* = 0.404, respectively). Mean window-specific foam height was highest at 43.37 ± 12.80 mm in the >10–18 h window, and pH declined monotonically from 4.69 to 3.94. Within each fermentation, the annotated frames were partitioned at frame level into a training and a validation subset only, with no held-out test subset and no pooled model; the class-wise evaluation recorded for nine of these per-batch models gave a mean intersection-over-union of 0.190 ± 0.077 across the classes each model used (six in eight cases, five in one) and 0.371 ± 0.226 for the foam class, so segmentation accuracy on fermentations unseen during training was not established. The 307 observations recorded beyond 72 h (26.8% of the dataset) lie outside the annotated temporal domain. The findings support the proof-of-concept feasibility of longitudinal, image-based foam characterisation under controlled laboratory conditions but do not establish absolute measurement accuracy, generalisation to fermentations unseen during training, or readiness for process-control deployment. The semantic-segmentation component was used as an image-analysis instrument for extracting longitudinal foam descriptors rather than as a newly proposed segmentation architecture. In a targeted experiment on a fixed batch-level partition, with three complete fermentations withheld for testing, replacing class-weighted cross-entropy with an asymmetric Tversky loss raised foam recall on sparse-foam frames from 0.074 to 0.318 without any change to the network architecture, whereas the tested attention-gated configuration, which adds skip connections and attention gates together, did not improve foam segmentation under weighted cross-entropy. Accordingly, the methodological contribution of the study resides in the longitudinal multi-batch acquisition design, integration of image-derived descriptors with synchronised physicochemical measurements, and batch-aware analysis of repeated fermentation observations. The present data do not establish architectural superiority, segmentation generalisation to unseen fermentations, or a technical advance in semantic-segmentation methodology.

## 1. Introduction

Supported by developments in deep learning, computer vision has emerged as a high-precision technology for detecting and characterising foam in bioreactor applications [1]. Integrating artificial intelligence into bioprocessing enables real-time data processing and predictive modelling, capabilities that are particularly relevant to the dynamic nature of fermentation processes [2].

Advances in computer vision and deep learning have substantially improved foam detection during alcoholic fermentation, with U-Net neural networks becoming the predominant architecture for automated segmentation [3]. The contribution of the present study lies neither in the segmentation architecture nor in a comparison of architectures, but in following image-derived foam descriptors longitudinally across twelve independent fermentations and analysing them with the fermentation batch as the experimental unit, a combination that, to our knowledge, has not previously been reported for longitudinal beer wort foam characterisation across multiple independent fermentation batches.

Foaming presents a challenge in industrial bioprocesses, including alcoholic fermentation in brewing, and requires reliable detection and control methods to prevent operational problems [1]. Carbon dioxide formed during the conversion of carbohydrates into ethanol generates foam [4]. Without suitable monitoring protocols, uncontrolled foaming may cause severe operational disruption, including reactor overpressurisation and loss of sterility [1].

Foam generated during fermentation is a well-documented phenomenon in beer wort fermentation. It consists of CO_2_ bubbles stabilised by surface-active compounds, including proteins and carbohydrates released by yeast cells at the gas–liquid interface [5]. The interplay between biological processes and the physical expression of foam creates a dynamic system in which fermentation by-products govern foam stability and structure.

Excessive foam formation can cause serious operational problems, such as filling the fermenter’s headspace, causing overflow and contamination, and resulting in product loss [5,6]. Beyond these immediate physical constraints, excessive foaming may affect yeast-cell metabolism and reduce productivity. In carbonated beverages such as beer, foam characteristics influence sensory attributes, including aroma release and taste perception [4].

Beer wort fermentation is subject to increasingly stringent process-optimisation requirements. Operational efficiency therefore depends on appropriate monitoring of foaming capacity and foam stability, including foam height, collapse rate, foam density, foaming duration and adhesion to the glass surface [4].

The accuracy of foam-prediction models [7] is influenced by variables in the fermentation environment, including pH, temperature, density and composition (carbohydrates, FAN and minerals). The initial substrate concentration and its changes during fermentation are critical to controlling nutrient flow and estimating the conversion stage [7], highlighting the need for adaptive monitoring systems that can accommodate fluctuating input conditions.

Conventional methods for quantifying foam are often labour-intensive, and many facilities rely on operators to monitor alcoholic fermentation visually and intervene manually. Established semi-automated methods include level sensors based on conductivity, impedance and laser technologies, as well as volumetric measurements [8]. These methods have practical limitations, provide restricted and potentially inaccurate information, and may not offer the repeatability required for consistent industrial control [4]. They are also time-consuming [6].

Alcoholic fermentation is complex, and variability in process conditions reduces measurement accuracy [9], creating a need for algorithms that can adapt to these changes.

Previous studies have highlighted the difficulties of optical foam detection under industrial conditions. Analysis based on standard RGB images is unreliable in practice because light reflections, variations in focus and foam adhesion to vessel walls impede accurate detection of the foam boundary [10]. Other approaches have used 3D cameras to obtain the foam’s three-dimensional profile and have shown greater robustness, enabling objective and repeatable measurement of foam stability [10].

Integrating machine learning with the science of alcoholic fermentation represents a shift from subjective manual assessment to automated, precise foam analysis [11]. Machine-learning models built on robotic-pourer image descriptors have reported correlation coefficients of about 0.9 with sensory-panel assessments [12,13], and automated deep-learning image analysis has been reported to extract bubble and foam descriptors substantially faster than conventional threshold-based image processing [3,14,15,16,17].

Machine-learning classifiers have been applied to sensor signals in order to infer the conditions inside industrial tanks, including the presence of foam and turbulence, from radar level-measurement curves [9]. That work reports good classification accuracy for the conditions considered and illustrates the usefulness of learned classifiers in industrial measurement settings [9]. Convolutional-neural-network models have also been embedded in low-cost imaging sensors that classify airlock images by the presence or absence of gas bubbles and aggregate those classifications into a cumulative index of fermentation activity [18].

U-Net performs strongly in foam and bubble segmentation because its encoder–decoder architecture and skip connections enable the accurate edge detection required for precise measurements [19,20].

Multivariate statistical process monitoring has been applied to the occurrence of foaming in batch fermentation. Multiway partial least squares models, fitted to three-dimensional datasets of batches, process variables and measurements, relate the process variables to a foaming indicator derived from the exhaust differential pressure, and distinguish variation within a batch from differences between batches [21].

Advanced learning techniques allow automatic measurement of foam volume, stability, and height, as well as bubble-size distribution and bubble count. RoboBEER, developed at the University of Melbourne, is the most extensively validated implementation. It measures more than 15 variables, including maximum volume, total foam duration, and drainage rate, and achieved 92.4% accuracy in identifying fermentation type [11,12,13,14,15,22].

Temporal-analysis capabilities have expanded through advanced neural architectures. Variational autoencoders have been used to generate synthetic fermentation time series that augment sparse experimental datasets and improve the predictive performance of soft sensors [23]; recurrent architectures such as long short-term memory networks are used more generally to model sequential process data.

AI-based foam-monitoring systems may have important economic implications, but these cannot be quantified without a cost–benefit analysis and validation under operational conditions. Multimodal systems combine computer vision with ultrasonic sensors, electronic-nose arrays, and chemical sensors to characterise the process comprehensively [17,24].

Computer vision combined with neural networks is used to assess bubble size and foaming capacity, both of which are related to fermentation quality [11,25]. By analysing video sequences or still images, these technologies can identify subtle changes in foam dynamics that may indicate contamination or changes in the composition of the fermentation medium.

Despite substantial progress in applying artificial intelligence to fermentation-foam monitoring, published work still rests predominantly on single fermentations or on pooled frame collections, so the ability of image-derived descriptors to generalise across independent fermentation runs remains largely uncharacterised. Current approaches also assume standardised fermentation conditions and treat individual frames as independent observations. What is therefore needed is a longitudinal, batch-aware characterisation of image-derived foam descriptors, acquired under controlled conditions across several independent fermentations and analysed at the level of the experimental unit.

Recent studies have demonstrated the feasibility of deep-learning-based image analysis for foam and bubble monitoring across fermentation and bioprocess systems, including foam detection in stirred-tank bioreactors [26] and foam segmentation in wastewater-treatment plants [6]; specifically for beer wort primary fermentation, a recent comparative benchmark evaluated optimised U-Net architectures against DeepLabV3+, Fast-SCNN and SegNet [27]. Accordingly, and as stated above, the intended contribution of the present study does not reside in the segmentation architecture itself but in the longitudinal application of image-derived foam descriptors across independent fermentations and in their integration with synchronised physicochemical records on a common time base. Because hourly frames are nested within batches, the statistical analysis treats the fermentation batch as the experimental unit.

This study therefore aimed to implement and evaluate a method for monitoring alcoholic fermentation by integrating convolutional-neural-network-based semantic segmentation with multiparametric sensor-data acquisition. The system was designed for at-line acquisition (Δt = 60 min) and for the morphological and dynamic characterisation of foam. The study was exploratory and addressed the proof-of-concept feasibility of the complete workflow—longitudinal acquisition, segmentation, descriptor extraction and batch-aware analysis—at laboratory scale, rather than the validated performance of the segmentation step or of the system under industrial conditions.

The study also addressed the following objectives:

- Multimodal data integration with image analysis: combined monitoring of visual data and physicochemical measurements (pH, temperature, and dissolved oxygen concentration).
- Methodological evaluation using beer wort at extract concentrations of 12, 15 and 18 °P, thereby covering a range relevant to industrial conditions.
- Methodological transparency through a documented image-processing and statistical pipeline, with between-batch consistency reported descriptively.
- Batch-aware statistical analysis of the resulting descriptors, in which the fermentation batch is treated as the experimental unit and observation-level correlations are reported as exploratory.

Accordingly, the present study does not propose a new semantic-segmentation architecture and does not claim methodological novelty at the level of neural-network design. The segmentation component serves as an image-analysis instrument within a broader fermentation-monitoring workflow. The scientific contribution addressed here is instead the longitudinal application of image-derived foam descriptors across twelve independent beer wort fermentations, their integration with synchronised physicochemical measurements on a common time base, and their analysis with the fermentation batch recognised as the experimental unit. This distinction is important because the study evaluates the feasibility and interpretability of a longitudinal monitoring workflow rather than the superiority of a particular computer-vision architecture. The underlying physicochemical mechanisms of foam formation were not measured and are not resolved by this design.

## 2. Materials and Methods

The study was conducted in the Research Laboratory for Modelling, Simulation and Advanced Control of Technological and Biotechnological Processes in the Food Industry, Faculty of Agricultural Sciences, Food Industry and Environmental Protection, Lucian Blaga University of Sibiu, Romania.

A controlled laboratory experimental design with repeated measurements over time was used to monitor the dynamics of alcoholic fermentation in beer wort. The initial extract concentration of the wort was the experimentally manipulated factor and was set at three levels: 12, 15 and 18 °P; all other operating conditions were intended to be held constant, and the physicochemical and image-derived variables were observed rather than manipulated. The departures from the nominal temperature setpoint that were actually recorded are reported in Section 2.2 and treated as a limitation in Section 4.4. The design was chosen to capture temporal changes in foam morphology and environmental variables throughout the fermentation cycle. The main objective was to implement and evaluate a computer-vision-based method for non-invasive, at-line (Δt = 60 min), image-based characterisation of fermentation foam.

The sample comprised 12 independent fermentations distributed among initial extract levels of 12 °P (*n* = 5), 15 °P (*n* = 4) and 18 °P (*n* = 3). This design was exploratory and was intended to characterise within-batch trajectories and between-batch variability. The 1147 hourly observations are repeated longitudinal measurements nested within the 12 fermentations, so the experimental unit for inferential comparisons was the fermentation batch (*n* = 12). No formal power analysis was performed before the experiments; this decision reflects the exploratory nature of the study, which sought to characterise foam dynamics using dense time series. This limitation was considered when interpreting inferential results. Comparisons among wort extract concentration levels (°P) were based on batch-level aggregate statistics to reduce temporal dependence among observations.

### 2.1. Beer Wort and Yeast

The beer wort was prepared from Brewferm Wheat Tripel concentrated wort (manufactured by Brewferm, Baverlo, Belgium, and distributed by BRICO IDEEA SRL, Bucharest, Romania). It was diluted with single-distilled water produced using a laboratory-scale distillation apparatus manufactured by BOECO, Hamburg, Germany; the water was obtained from the municipal drinking-water supply of Sibiu, Romania (≤5° dH). The three target extract concentrations were 12, 15 and 18 °P. Wort extract concentration and pH were determined before and after fermentation in accordance with the Analytica-EBC Methods.

Beer wort fermentation used the easy-to-use (E2U™) top-fermenting yeast strain SafAle™ S-33 (taxonomy: *Saccharomyces cerevisiae*; apparent attenuation: 68–72%; pitching rate: 50–80 g/hL; fermentation temperature range: 18–26 °C; alcohol tolerance: 9–11%; phenolic off-flavour: negative; medium sedimentation, without clumping and dispersing as a fine haze after resuspension). SafAle™ S-33 is a commercial active dry yeast (ADY) produced by Fermentis-Lesaffre S.A. (Marcq-en-Barœul, France) and is suitable for Tripel-style wheat beer. Each fermentation was inoculated with 1.0 g of active dry yeast, sprinkled directly onto the wort surface without prior rehydration, as described for E2U™ direct pitching in the manufacturer’s technical data sheet for SafAle™ S-33. For the 1.5 L working volume, this is 66.7 g/hL, within the 50–80 g/hL recommended in that data sheet, and at the specified viability of more than 1.0 × 10^10^ cfu/g, it corresponds to more than 6.7 × 10^6^ viable cells per millilitre of wort. The same yeast mass was used in every fermentation, so the nominal mass-based pitching rate was constant between batches; the viability of each individual charge was not measured separately. The nominal fermentation-temperature setpoint was 20 °C, within the 18–26 °C range recommended for this strain.

Certified buffer solutions (pH 4.01 ± 0.01, 7.00 ± 0.01 and 10.01 ± 0.01 at 25.0 °C) traceable to the standards of the National Institute of Standards and Technology (NIST, Gaithersburg, MD, USA) were used for 3-point calibration of the pH meter.

The pO_2_ sensor was calibrated using a zero-oxygen solution (2% *w*/*v* sodium sulphite in ultrapure water; resistivity 18.2 MΩ·cm; Milli-Q system, Merck Millipore, Darmstadt, Germany) and air-saturated water at 20.0 ± 0.1 °C. Sensor performance was checked at 72 h intervals, with an accepted tolerance of ±2% relative to the reference value.

Temperature was calibrated using an NIST-certified mercury thermometer (accuracy ±0.05 °C, traceable to national metrological standards). Thermal stability was monitored continuously with a target tolerance of ±0.1 °C; the observed stability is reported separately in Table 1.

Spatial calibration of the imaging system used: (i) a neutral grey card with certified reflectance of 18.0 ± 0.5% (X-Rite ColorChecker, Grand Rapids, MI, USA) to standardise white balance; and (ii) precision graph paper with a stated dimensional tolerance of ±0.1 mm to determine the pixel-to-millimetre conversion factor.

### 2.2. Fermentation System

Fermentations were conducted in a 2 L glass bioreactor operated in batch mode (working volume 1.5 L) equipped with online systems for measuring and controlling temperature, pH, pO_2_, level and rotational speed, together with BIOSCADA supervisory control and data-acquisition software. Batch mode is meant here in the strict sense: no substrate, nutrient or base was fed after inoculation, and no gas was sparged into the medium at any point during fermentation. The wort was not aerated after pitching; the vessel remained closed, and the carbon dioxide generated by fermentation was vented through a one-way valve in the lid. With 1.5 L of wort in the 2 L vessel, the gas headspace above the liquid was approximately 0.5 L, that is, about one-quarter of the vessel volume. The bioreactor had a stainless-steel lid fitted with sensor ports. The nominal temperature setpoint was 20 °C, controlled by a proportional–integral–derivative (PID) controller (accuracy ± 0.05 °C) integrated with an external water bath set to 15 ± 0.1 °C. Agitation was maintained automatically at 150 ± 2 RPM by an impeller positioned 5.0 cm above the bottom of the vessel. Precise PID temperature control was selected because fermentation temperature has a documented effect on the course and the rate of beer fermentation [28]. The recorded process values did not remain within the controller-accuracy band. Across the 11 batches, excluding the batch containing the two isolated extremes, observed batch ranges extended from 19.20 to 25.32 °C. In the remaining batch, isolated readings of 27.5 °C at the initial acquisition and 11.5 °C at a later acquisition were adjacent in the series to values of approximately 21.6–21.8 °C. In the absence of an independent reference logger, the available records do not permit a distinction between transient acquisition or sensor deviations and actual short-term process fluctuations. All recorded values are reported (Table 1), and the departures from the nominal setpoint are treated as a limitation because fermentation temperature affects fermentation kinetics and may therefore also affect the foam dynamics recorded here. The PID configuration used for bioreactor temperature regulation follows previously described practice [29].

### 2.3. Image-Acquisition Hardware

The image-acquisition system included a Logitech BRIO Ultra HD Pro digital camera (Logitech International S.A., Lausanne, Switzerland) with a native resolution of 4096 × 2160 pixels, 24-bit colour depth and spectral sensitivity over 400–700 nm. The camera was mounted on a rigid support 10.0 ± 0.1 cm from the side wall of the fermentation vessel to ensure reproducible acquisition geometry.

The experimental assembly was housed in an enclosure whose inner walls were coated with matte black paint (reflectance < 5% in the visible range) to minimise stray reflections and exclude external light. This approach was used to optimise the signal-to-noise ratio in bioanalytical imaging.

Standardised illumination was provided by a 50 cm white LED strip (colour temperature 5000 ± 200 K; colour rendering index (CRI) > 95), positioned symmetrically around the vessel to ensure photometric uniformity and eliminate direct shadows. The hourly, synchronised switching of the lighting circuit was controlled by an Arduino UNO microcontroller module (Arduino LLC, Somerville, MA, USA) programmed to maintain consistent optical conditions throughout the experiment.

### 2.4. Computing Infrastructure and Software Environment

Data processing, deep-learning model training and inference were performed on a high-performance workstation configured with an Intel Core i7-13620H processor (base frequency 2.4 GHz, 24 MB L3 cache, up to 4.9 GHz in Turbo mode and 10 processing cores), a dedicated nVIDIA GeForce RTX 4060 graphics card with 8 GB GDDR6 memory and 64 GB DDR4-3600 RAM.

The principal software environment was MATLAB R2024a (MathWorks Inc., Natick, MA, USA), the release recorded in the header of the archived training script, with the Deep Learning Toolbox (version 24.1), Computer Vision Toolbox (version 24.1) and Image Processing Toolbox (version 24.1) used to develop and implement the computer-vision algorithms. The trained model files are dated December 2025.

No second deep-learning framework was used. Every step that produced the results reported here—annotation, network training, inference and morphometric feature extraction—was implemented in MATLAB with the toolboxes named above. Most of the descriptive and inferential statistical analysis of the extracted descriptors was performed in Python 3.13, whereas the linear mixed-effects sensitivity analysis reported in Section 3.3 was fitted in MATLAB R2024a with fitlme; the versions and libraries are given in Section 2.5.4.

### 2.5. Experimental Protocols

#### 2.5.1. Fermentation Conditions

Apart from the initial wort extract level, the fermentation protocol was identical for every batch, subject to the recorded temperature deviations reported in Section 2.2. The pH of the medium was not actively controlled after inoculation, allowing the natural dynamics associated with yeast metabolic activity to be observed.

Automated process monitoring recorded the following variables at 60 min intervals: temperature (±0.1 °C), pH (±0.02 units) and dissolved oxygen concentration, expressed as pO_2_ (% air saturation) (±2% of full scale), measured in the liquid phase using an immersed probe.

#### 2.5.2. Synchronised Image-Acquisition Protocol

Standardised data acquisition

Image acquisition was synchronised to maintain optimal data quality throughout fermentation. Acquisition began at yeast inoculation (t_0_) and continued at regular intervals of 60.0 ± 0.5 min, consistent with approaches reported for at-line monitoring using imaging and multivariable sensors [30].

Images were saved in PNG format at the acquisition resolution (1920 × 1080) with the acquisition timestamp encoded in the filename, then resized to 512 × 512 pixels to improve the efficiency of AI-assisted processing [30,31].

Environmental variables were monitored using calibrated sensors for temperature (accuracy ± 0.1 °C), pH (accuracy ± 0.02 pH units) and pO_2_ (accuracy ± 2%), sampled every 60 min in synchrony with image acquisition.

Data collected from the Sartorius module were saved in .txt format and included the acquisition date and time, fermentation time, temperature (°C), pH and pO_2_ (%). A dedicated Python script was implemented to integrate these measurements with the visual data.

Development and within-batch evaluation of the segmentation component

(a) Segmentation model and training

A convolutional encoder–decoder network was implemented in MATLAB R2024a using the Deep Learning Toolbox and Computer Vision Toolbox.

Dataset preparation included resizing the images to 512 × 512 pixels. Training frames were augmented online with random rotations (±20°), random horizontal and vertical reflections, random scaling (0.8–1.2), random shear (±10° in both axes) and random brightness jitter (0.8–1.2×). The encoder comprised three convolutional levels with 64, 128 and 256 filters (3 × 3 convolutions, batch normalisation, ReLU, dropout of 0.3, 0.3 and 0.4, and 2 × 2 max pooling after the first two levels). The decoder comprised two levels of 2 × 2 transposed convolutions (128 and 64 filters), each followed by a 3 × 3 convolution, batch normalisation and ReLU. A 1 × 1 convolution and a softmax layer produced the per-class maps, and the output was a pixel-classification layer with inverse-frequency class weights. Inspection of the archived networks shows that this weighting was in force in nine of the twelve per-fermentation models; in the remaining three (13 March 2025, 22 May 2025 and 22 July 2025), the class-weighting field is set to ‘none’, so those models were trained without class weighting. The network contained no skip connections between encoder and decoder and no pre-trained encoder; all weights were initialised randomly.

(b) Training configuration and optimisation

The network was trained with the Adam optimiser at an initial learning rate of 1 × 10^−4^, reduced by a factor of 0.5 every 5 epochs, with a mini-batch size of 2 frames, L2 regularisation of 0.001, shuffling at every epoch, validation every 5 iterations and a validation patience of 20, for a maximum of 100 epochs. The loss was the multi-class cross-entropy of the pixel-classification layer, class-weighted in the nine models for which weighting was in force and unweighted in the remaining three.

(c) Sampling and annotation strategy

To obtain adequate morphological representation, a stratified sampling strategy over fixed acquisition intervals was applied, with a target allocation of approximately 20% of the annotated frames from the early interval (0–6 h), 50% from the intermediate interval (>6–36 h) and 30% from the late interval (>36–72 h). These are intervals defined on the acquisition time axis and not batch-specific physiological transitions. This allocation reflects the importance of varied, well-labelled datasets for robust training of deep-learning models [26].

(d) Image preprocessing

Images were preprocessed in MATLAB using the following standardised steps: resizing to 512 × 512 pixels, defining a region of interest (ROI) from a reference image, applying the ROI to the complete dataset, and organising the processed images in structured directories.

(e) Data labelling and annotation

Pixel-level semantic annotation was performed for 223 uniquely referenced frames drawn from all 12 fermentations, corresponding to 19.4% of the 1147 acquired frames, with 14 to 24 annotated frames per batch; the annotated frames were distributed across extract levels as 92 frames from 5 batches at 12 °P, 75 frames from 4 batches at 15 °P and 56 frames from 3 batches at 18 °P (Table S2). Within the archive, these 223 frames are held as two inventories: 209 frames from 11 fermentations, catalogued as the primary annotation inventory, and a further 14 frames annotated for the twelfth fermentation (1 July 2025, 18 °P) and used only for that fermentation’s own model. The distinction is one of archival provenance and has no analytical consequence because every network was trained on the annotated frames of its own batch alone; 223 frames from 12 fermentations is therefore the figure used whenever the annotated dataset as a whole is described, and the 209 + 14 split is retained only here and in Table S2 for traceability to the archive. All annotated frames were acquired within the first 72 h of fermentation. Labelling was performed using the Image Labeler app of the Computer Vision Toolbox version 24.1 (MATLAB R2024a), which provides polygonal region labelling. Model development was carried out per fermentation rather than on a pooled dataset: a separate network was trained for each of the 12 batches on the annotated frames of that batch alone, which were partitioned at frame level by random permutation into a training subset (80%) and a validation subset (the remainder). Across all 12 fermentations, the overall totals were 177 training and 46 validation frames, of which 166 and 43, respectively, belong to the 209-frame primary inventory (Table S3). No held-out test subset was defined for any batch, the partition was neither stratified nor grouped, no random seed was fixed, and no model was trained on frames pooled across fermentations. Segmentation accuracy was therefore never quantified on a fermentation that had not contributed frames to the model being evaluated.

Label taxonomy: the annotation system used a hierarchical taxonomy based on the temporal and functional characteristics of the elements identified in the field of view (Figure 1), clearly distinguishing the dynamic components of fermentation from the static elements of the experimental system.

Seven label categories were annotated. The intended training taxonomy comprised six pixel classes (foam, foam-free exposed area, residual gas, liquid, metal and background); the spatial calibration marker served as a dimensional reference rather than as a training class. Inspection of the archived per-fermentation networks shows that this taxonomy was not applied uniformly across the twelve models: eight were trained on all six classes, the model for 19 March 2025 on five (the metal class absent) and the models for 13 March 2025, 22 May 2025 and 22 July 2025 on four (metal and background absent). The pooled experiment described in Section 2.5.3 applies the full six-class taxonomy uniformly to all 223 annotated frames and is therefore not configured identically to the per-fermentation models.

The dynamic segmentation classes were as follows:

- Foam region (Figure 1a): The active foam layer was delineated using high-precision polygonal contours (yellow in the visualisation system). This class captures the complex gas–liquid interface characteristic of fermentation, including variation in bubble morphology and the non-uniform structure of the foam layer;
- Foam-free exposed area (Figure 1b): The liquid surface/interfaces that became visible as the foam layer receded were delineated with brown polygons, allowing the progressive exposure of the free surface during fermentation to be quantified. This class is not a chemical measure of oxidation but an imaging proxy for the area not covered by foam.

The static segmentation classes were as follows:

- Metal components (Figure 1c): Stainless-steel structural elements (impellers, sensors and supports) were segmented using light-blue polygons. This allowed these regions to be excluded from the morphological analysis of foam and prevented detection artefacts;
- Residual gas spaces (Figure 1d): Air voids trapped within the liquid structure or at solid–liquid interfaces were delineated using white polygons. This distinguished them from active bubbles in the foam layer and kept them outside the foam class during segmentation;
- Background elements (Figure 1e): Visual components outside the fermentation system were identified using orange polygons, enabling subsequent application of background-subtraction algorithms and improving the signal-to-noise ratio during image processing;
- Liquid phase (Figure 1f): The malt wort visible beneath the foam layer was delineated using turquoise polygons, providing a separate semantic class for the bulk liquid so that it was not confused with the foam layer or with the foam-free exposed area;
- Spatial calibration marker (Figure 1g): A dimensional reference element of known length (15.0 mm) was marked with a yellow line. The pixel-to-millimetre conversion factor required for all morphometric measurements was obtained by manually selecting two points on this reference in a reference frame of each acquisition series.

Annotation quality-assurance protocols: The labelling process incorporated several levels of quality control. The annotations were independently reviewed by three annotators experienced in the analysis of fermentation images. Agreement among the three annotators was reviewed qualitatively as labelling proceeded; no per-frame agreement coefficient was computed or archived, so none is reported. Ambiguous cases were resolved through iterative review and consensus. Decision criteria for borderline cases were documented systematically to maintain consistency across the dataset.

Development and per-batch training of the segmentation models

The convolutional encoder–decoder network described above was implemented in MATLAB R2024a with the Deep Learning Toolbox and Computer Vision Toolbox, and one instance of it was trained for each fermentation on that fermentation’s own annotated frames. Inference for a given fermentation was then performed with the model trained on that same fermentation. This per-batch scheme amounts to a supervised re-calibration of the segmentation step for every run; it does not produce a single model intended to generalise to new fermentations, and the consequences for the interpretation of all downstream descriptors are set out in Section 3.1 and Section 4.1.

Morphological foam characteristics

After model training, inference was performed on the complete set of images captured during fermentation. The feature-extraction module implemented image-analysis algorithms to derive several descriptors from each segmented frame, supporting at-line monitoring and characterisation of fermentation processes [32].

The principal foam characteristics were as follows:

- Foam height (mm): Calculated by analysing the vertical intensity profile, with the upper and lower boundaries detected using peak-detection algorithms applied to the row-integrated profile. Subpixel refinement was performed by parabolic interpolation to improve precision;
- Foam-free exposed area (mm^2^): Obtained by counting the pixels classified as foam-free exposed area within the valid region of interest and converting that pixel count to metric units using the experimentally determined pixel-to-millimetre spatial calibration factor.

In the image analysis, the term ‘frame’ denotes an individual image acquired at a specific time during fermentation and represents the spatial domain available for analysis. By contrast, a segmented class area is the portion of the valid region of interest (ROI) assigned by segmentation to one pixel class. The frame is therefore the imaging unit of observation, whereas a segmented class area is a variable derived from that frame. The foam-free exposed area reported here was determined in mm^2^ after spatial calibration of the image; expressed relatively, it is the proportion of the total analysed ROI segmented as foam-free exposed area. Regions outside the valid analysis area were excluded from the calculation.

#### 2.5.3. Targeted Experiment on Class Imbalance

To test directly whether the class imbalance identified in the archived evaluation can be mitigated, a separate segmentation experiment was carried out on the annotated frame set. Unlike the per-fermentation models used to derive the foam descriptors reported in Section 3, this experiment used a single pooled model and a fixed partition defined at the level of complete fermentations, so that the effect of a loss function and of an architectural modification could be estimated independently of partition variability. The experiment characterises the segmentation component; it does not regenerate the descriptors analysed in Section 3.

All 223 annotated frames and their six-class ground-truth masks were pooled. Complete fermentations were assigned to training (seven batches, 142 frames), validation (two batches, 28 frames) and testing (three batches, 53 frames) using a fixed random seed, with the test batches stratified so that one fermentation from each initial extract level was withheld (9 April 2025, 12 °P; 26 March 2025, 15 °P; 2 April 2025, 18 °P). No frame from a test fermentation contributed to training or validation. Pixels not covered by any annotation polygon, which account for 21.7% of all pixels, were excluded from both the loss and the evaluation metrics.

Four configurations were trained in a 2 × 2 factorial design combining two architectures with two loss functions. The first architecture reproduced the archived convolutional encoder–decoder network. The second added encoder-to-decoder skip connections and additive attention gates while retaining the same depth and filter widths (64/128/256, two pooling levels), so that the contrast is not confounded by network capacity. Because the skip connections and the attention gates were introduced together, this configuration is evaluated as a whole, and the contribution of the attention gates cannot be separated from that of the skip connections; separating them would require a further configuration with skip connections but without gating, which was not trained. The two loss functions were class-weighted cross-entropy, with weights derived by inverse frequency from the training batches only, and the Tversky loss with the false-negative term weighted at 0.7 and the false-positive term at 0.3, an asymmetry that penalises missed pixels of rare classes.

Every other element was held identical across the four runs: the data, the partition, the random seed, the augmentation pipeline, the Adam optimiser (initial learning rate 1 × 10^−3^, halved every 20 epochs), the mini-batch size of four, L2 regularisation of 1 × 10^−3^, an input resolution of 256 × 256 pixels, early stopping with a patience of ten validations, and selection of the network with the lowest validation loss. Training was performed in MATLAB R2026a on a central processing unit. Performance was assessed on the three withheld fermentations as intersection-over-union, Dice coefficient, recall and precision, both aggregated over the pooled confusion matrix and per frame. Per-frame foam intersection-over-union values were compared between configurations with the Wilcoxon signed-rank test, with 95% percentile bootstrap confidence intervals from 2000 replicates and effect sizes reported as r = |Z|/√N. Because every test frame originates from only three independent fermentations, this is a paired frame-level analysis conditional on those three batches rather than inference over independent experimental units; it is reported as technical support for the comparison, alongside the direction of the effect in each withheld fermentation considered separately.

#### 2.5.4. Statistical Analysis

Image acquisition, segmentation and morphometric feature extraction were performed in MATLAB R2024a (MathWorks, Natick, MA, USA) using the Deep Learning, Computer Vision and Image Processing Toolboxes (all version 24.1). Descriptive statistics, non-parametric tests and correlation analyses were performed in Python 3.13 with pandas 2.2.3, numpy 2.2.6, scipy 1.16.1 and statsmodels 0.14.6, with the random seed fixed at 42. The linear mixed-effects sensitivity analysis described below was fitted in MATLAB R2024a with fitlme (Statistics and Machine Learning Toolbox version 24.1), so the statistical analysis reported here used both environments. The longitudinal dataset was obtained from 12 independent fermentations. Inferential comparisons among conditions, such as wort extract concentration levels (°P), used batch-level aggregate statistics to reduce temporal dependence among observations; analyses at the level of individual temporal observations were considered exploratory. The dataset included physicochemical variables monitored over time (temperature, pH and pO_2_) and foam-morphology indicators extracted automatically by image analysis. Data processing, statistical testing and modelling used standard library functions together with custom routines written for this study.

Complete descriptive statistics were calculated for each monitored variable, including the mean, standard deviation (SD), coefficient of variation (CV%), minimum and maximum values, quartiles (Q1, median and Q3), interquartile range (IQR), skewness, and kurtosis. Distribution normality was assessed using the Shapiro–Wilk test (α = 0.05) [33]. Given the temporal structure of the data and the frequent departures from normality observed for most variables, the descriptive and exploratory group comparisons were performed with non-parametric methods; the principal batch-aware inference is the linear mixed-effects sensitivity analysis described at the end of this section and reported in Section 3.3.

Variables were compared among independent groups (initial wort extract concentration levels, °P, and operational time windows) using the Kruskal–Wallis test [34], while the Friedman test was applied to repeated measurements from the same batches.

Relationships among continuous variables were analysed using the Pearson correlation coefficient (r), calculated at the level of individual hourly observations (*n* = 1147) to characterise approximately linear associations on an exploratory basis. To assess the robustness of associations involving fermentation time to within-batch temporal dependence, these associations were also recalculated using batch-level aggregated data; that reassessment excluded the 7 May 2025 fermentation, whose source file carries no directly logged fermentation-time column, and therefore rests on 11 batches (Section 3.1). Observation-level correlations are reported as exploratory descriptions of covariation and not as inferential tests at the level of the experimental unit. Statistical significance was set at *p* < 0.05. For the five batch-level comparisons among extract concentration levels reported in Section 3.2.4, Bonferroni correction gave a corrected α = 0.05/5 = 0.0100 [35].

Data preprocessing included identifying outliers using the interquartile-range criterion (±1.5 × IQR) [36]. Missing-data handling followed standard practice for experimental time series [37]: the final analytical dataset was checked and contained no missing values in any monitored variable across the 1147 observations, so no imputation was performed for any result reported here. Logarithmic transformations were applied before parametric analysis to variables with log-normal distributions.

Definition of the operational time windows: the six windows used in the statistical analyses (Lag, Growth, High Kräusen, Decline, Conditioning and Final) were defined by fixed time thresholds measured from inoculation, established independently of the foam-morphology descriptor values subsequently tested. The thresholds applied to the analysed dataset were Lag (0–3 h), Growth (>3–10 h), High Kräusen (>10–18 h), Decline (>18–40 h), Conditioning (>40–55 h) and Final (>55 h); the same thresholds were applied to every batch, and every observation was assigned to exactly one window. These thresholds place the mean time to peak foam height (16.34 ± 5.34 h; Section 3.2.6) inside the High Kräusen window. They are distinct from, and should not be confused with, the coarser three-interval scheme (0–6 h, 6–36 h and 36–72 h) used earlier only to stratify the sampling of frames for annotation. Because the actual time to peak foam height varied considerably among batches (6.00–26.79 h; Section 3.2.6), these windows are fixed operational time intervals that approximate the typical group-level metabolic pattern rather than biological phases independently confirmed for every batch. The conventional names (Lag, Growth, High Kräusen, Decline, Conditioning and Final) are retained as labels for these temporal windows by approximate analogy with established fermentation stages.

The Friedman test for repeated measures was used to assess changes in monitored variables across successive operational time windows. The experimental batch was treated as the repeated observational unit, and operational time window was the within-batch factor. The analysis was applied to the physicochemical and morphological variables monitored during fermentation, including pH, temperature, pO_2_, foam height and foam-free exposed area.

The Friedman test was selected as a non-parametric alternative for comparing dependent measurements because of the longitudinal data structure and the departures from normality identified for some variables. The Friedman χ^2^ statistic was calculated for each variable, and statistical significance was assessed at α = 0.05.

The magnitude of concordance in variation across operational time windows was quantified using Kendall’s coefficient of concordance (W), calculated from the Friedman statistic. Values of W close to 0 indicate low concordance in the pattern of variation among batches, whereas values close to 1 indicate high concordance. This measure was used alongside the *p* value to assess the relevance and consistency of effects associated with operational time window.

Medians for each operational time window were calculated to describe the development of every variable. Relative changes among operational time windows were expressed as percentages of the median value in the reference window, facilitating interpretation of the magnitude of variation during fermentation.

To address clustering and temporal pseudoreplication, a sensitivity analysis used linear mixed-effects models after aggregating each outcome within fermentation batch and operational time window. Foam height and foam-free exposed area were transformed as log(1 + outcome). Each model included operational time window and initial extract level as fixed effects and fermentation batch as a random intercept and was fitted by restricted maximum likelihood with fitlme (Statistics and Machine Learning Toolbox); denominator degrees of freedom for the fixed-effect F tests were obtained by the Satterthwaite approximation. The analysis included all 12 fermentations and comprised the 70 non-empty cells of the 12 × 6 batch-by-window design; two cells were empty because one batch contributed no observation to the Growth window and another none to the High Kräusen window. For the 7 May 2025 fermentation, the window assignment used the reconstructed hourly sequence validated against the image time stamps (Section 3.1). The aggregation script, the aggregated input table and the model script are available from the corresponding author on reasonable request, subject to institutional approval and applicable confidentiality requirements, so that the values in Table S4 can be re-derived directly.

## 3. Results

### 3.1. Dataset and Annotation Coverage

The analysed dataset comprised 1147 hourly frames from 12 independent fermentations. The number of frames per fermentation was not equal (67 to 120) because acquisition ended at a different point in each run. Eleven of the twelve batch files carry a logged fermentation-time column; in the twelfth (7 May 2025), that column is absent, and fermentation time was reconstructed as the 0-based hourly index, which the image time stamps confirm to be spaced exactly 60 min apart. Observation-level analyses therefore used all 12 fermentations. The batch-level Pearson reassessment of the correlations involving fermentation time excluded the 7 May 2025 fermentation and consequently used 11 batches because that file lacked a directly logged fermentation-time column. The batch-by-window mixed-effects analysis reported in Section 3.3 included all 12 fermentations using the timestamp-validated reconstructed hourly sequence for 7 May 2025. Pixel-level ground-truth annotation covered 223 frames from all 12 fermentations, corresponding to 19.4% of all acquired frames, with 14 to 24 annotated frames per batch and a distribution across extract levels of 92 frames (5 batches) at 12 °P, 75 frames (4 batches) at 15 °P and 56 frames (3 batches) at 18 °P (Table S2). Of these, 209 frames from 11 batches constitute the primary annotation inventory of the archive, and 14 frames belong to the twelfth fermentation; that split is documented in Section 2.5 and Table S2 for archival traceability only. All annotated frames originated from the first 72 h of fermentation, and 307 of the 1147 observations (26.8%) were recorded after that limit. Model development was performed per fermentation: 12 separate networks were trained, each on the annotated frames of one batch, split at frame level into 80% for training and the remainder for validation (177 training and 46 validation frames across all 12 fermentations, of which 166 and 43, respectively, belong to the 209-frame primary inventory; Table S3). No batch was withheld from the training of the model subsequently applied to it, and no held-out test subset was defined, so segmentation accuracy was not quantified on any fermentation independent of model development.

A class-wise evaluation of the per-batch models was recorded for 9 of the 12 fermentations (Table S5). Averaged over the classes each model actually used—six for eight of the nine evaluated networks and five for 19.03.2025—accuracy was 0.380 ± 0.133 (range 0.164–0.576), and intersection-over-union was 0.190 ± 0.077 (range 0.081–0.323). For the foam class specifically, accuracy was 0.690 ± 0.285 and intersection-over-union 0.371 ± 0.226, with values spanning 0.000–0.739; for the foam-free exposed-area class, accuracy was 0.672 ± 0.243 and intersection-over-union 0.326 ± 0.164. In one fermentation, the foam class was not recovered at all (accuracy and intersection-over-union both 0.000). These values are low and highly variable, which is consistent with each model having been trained on only 11 to 19 frames, and they were obtained on frames from the same fermentation as the training data, so they are optimistic relative to any estimate that would be obtained on an unseen batch. Neither a pooled model nor a cross-batch evaluation exists for this dataset. Accordingly, the foam descriptors reported in the following sections are treated throughout as relative, within-batch indicators of foam dynamics, and no absolute measurement accuracy is claimed for them.

### 3.2. Characterisation of Fermentation-Process Variables

#### 3.2.1. Parameter Dynamics by Operational Time Window

Analysis by operational time window showed distinct profiles for the monitored variables. The 1147 observations were distributed as follows: Lag (*n* = 43; 3.7%), Growth (*n* = 76; 6.6%), High Kräusen (*n* = 81; 7.1%), Decline (*n* = 263; 22.9%), Conditioning (*n* = 179; 15.6%) and Final (*n* = 505; 44.0%). Window-specific differences in the image-derived foam-height estimate and the image-derived foam-free exposed-area estimate, stratified by the 12 °P, 15 °P and 18 °P levels, are summarised in Figure 2. For readability, the shorter forms ‘foam height’ and ‘foam-free exposed area’ are used from this point onwards, but both denote image-derived estimates whose absolute accuracy was not established (Section 3.1).

The window labelled Lag had the lowest values for the morphological foam variables. Foam height was 6.28 ± 7.04 mm, and the foam-free exposed area was minimal (18.62 ± 83.06 mm^2^). Mean pH was highest in this window (4.69 ± 0.20), while temperature was 21.79 ± 1.78 °C and pO_2_ was 9.45 ± 5.64% (Figure 2a,c).

In the Growth window, all morphological variables increased. Foam height rose to 30.40 ± 15.26 mm, approximately 4.8× the value in the Lag window, and the foam-free exposed area reached 478.80 ± 458.46 mm^2^. The pH decreased to 4.50 ± 0.18 (Figure 2a,c). Temperature was 21.80 ± 1.67 °C and pO_2_ was 10.29 ± 5.79%.

The maximum foam height (43.37 ± 12.80 mm) occurred in the operational time window conventionally labelled High Kräusen (>10–18 h) (Figure 2a). The foam-free exposed area reached 1040.54 ± 607.62 mm^2^ (Figure 2c). The pH continued to decline to 4.17 ± 0.19, a decrease of 0.52 units relative to the Lag window. Temperature was 21.67 ± 1.98 °C and pO_2_ was 9.71 ± 5.06%.

During the Decline window, foam height decreased to 31.15 ± 11.68 mm (Figure 2a). The foam-free exposed area increased to 2553.24 ± 1610.36 mm^2^ (Figure 2c). The pH decreased to 3.97 ± 0.08, a reduction of 0.72 units relative to the Lag window. pO_2_ was 9.27 ± 5.33%, and temperature was 21.62 ± 1.24 °C.

The Conditioning window showed a progressive reduction in foam height to 15.85 ± 6.42 mm, accompanied by an increase in the foam-free exposed area to 5659.19 ± 2104.07 mm^2^ (Figure 2a,c). The pH stabilised at 3.94 ± 0.08 and the temperature at 21.45 ± 1.12 °C. pO_2_ decreased to 8.68 ± 5.40%.

In the Final time window (>55 h, only partly represented among the annotated frames; see Limitations), foam height returned to a value comparable to that in the Lag window (6.62 ± 4.35 mm). The foam-free exposed area reached its maximum value of 9054.13 ± 2415.89 mm^2^, reflecting the greatest exposure of the liquid surface after dissipation of the foam layer (Figure 2c). The pH stabilised at 3.94 ± 0.07, temperature at 21.49 ± 0.98 °C and pO_2_ at 8.36 ± 5.05%. The overall change in pH across fermentation was 0.75 units, from 4.69 in the Lag window to 3.94 in the Final window.

The Friedman test identified statistically significant differences among operational time windows for most variables. The pH showed the most consistent pattern of window-related variation (χ^2^ = 47.89; *p* < 0.001; W = 0.958), indicating strong agreement among batches in the development of pH during fermentation. Median pH changed from 4.73 in the Lag window to 4.15 in the High Kräusen window and subsequently stabilised at 3.94 in the Final window, an overall decrease of 0.79 pH units (ΔpH = −0.79). Foam height also showed strong agreement among batches (χ^2^ = 44.91; *p* < 0.001; W = 0.898), with maximum values in the High Kräusen window. Median foam height increased from 5.04 mm in the Lag window to 41.68 mm in the High Kräusen window, approximately 8.3 times the initial value, and then decreased to 5.53 mm in the Final window. Differences in pO_2_ among windows were statistically significant (χ^2^ = 23.49; *p* < 0.001), but agreement was only moderate (W = 0.470), suggesting greater between-batch variability in dissolved-oxygen dynamics. The foam-free exposed area showed the most consistent window-related pattern of all the variables tested (χ^2^ = 50.00; *p* < 0.001; W = 1.000): its batch-level rank order across the six windows was identical in all 10 batches with complete window coverage, as expected for a variable that increased monotonically as the foam layer receded. Temperature was the only variable for which differences among windows did not reach statistical significance (χ^2^ = 6.74; *p* = 0.241; W = 0.135), with a median window-specific range of 21.45–22.00 °C; the departures from the nominal setpoint are reported in Section 2.2. Across the four variables with a statistically significant window effect, Kendall’s coefficient of concordance ranged from 0.470 to 1.000.

#### 3.2.2. Between-Batch Variability in the Fermentation Process

Process reproducibility was assessed by analysing variability among the means of the 12 experimental batches (Table 2). The between-batch coefficient of variation for temperature was 5.44% (overall mean: 21.62 °C; between-batch SD: 1.18 °C), indicating variability comparable to within-batch temporal fluctuation.

The pH had the lowest between-batch variability of all the monitored variables, with a CV of 1.92% (mean: 4.03; SD: 0.08).

pO_2_ (%) had the greatest between-batch variability (CV = 58.35%; overall mean: 8.47%; between-batch SD: 4.94%). This high variability may reflect the interaction between fermentation vigour (the rates of CO_2_ production and metabolic oxygen consumption by yeast) and the temperature-dependent kinetics of oxygen solubility and diffusion; neither was measured directly.

For the morphological foam parameters, the between-batch coefficients of variation were as follows: foam height, CV = 23.75% (mean: 18.15 mm; SD: 4.31 mm); and foam-free exposed area, CV = 29.57% (mean: 5439.12 mm^2^; SD: 1608.15 mm^2^). Yeast strain and the nominal mass-based pitching rate were held constant and were therefore not intentionally varied between batches; this may instead reflect differences in wort composition (protein and polypeptide contents), in the physiological state of the pitched yeast, and in operating conditions among batches.

#### 3.2.3. Correlation Analysis Among Process Parameters

Exploratory observation-level correlation analysis identified associations of different magnitudes among the variables monitored during fermentation. The strongest positive linear association was between fermentation time and foam-free exposed area (Pearson r = 0.876; *p* < 0.001), indicating a progressive increase in the area not covered by foam as fermentation proceeded.

By contrast, no relevant linear association was identified between pO_2_ and foam height (Figure 3; Pearson r = −0.034; *p* = 0.245; *n* = 1147). The Spearman correlation was positive but very weak (ρ = 0.090; *p* = 0.002), indicating that the statistical significance of this monotonic association was accompanied by a small effect size. Variation in pO_2_ therefore explained only a negligible proportion of the variation in foam height over the experimental range.

Foam height was also negatively associated with foam-free exposed area (r = −0.676; *p* < 0.001). This relationship indicates inverse covariation between the two variables during fermentation but does not show that development of the foam layer directly causes a reduction in the foam-free exposed area. Part of the association may reflect the shared temporal development of the two variables across operational time windows.

Fermentation time was negatively associated with both pH (r = −0.574; *p* < 0.001) and foam height (r = −0.686; *p* < 0.001). The inverse relationship between time and pH is compatible with the use of pH as an indicator of fermentation progress but does not in itself constitute independent validation of pH as a process marker. Similarly, pH was negatively associated with foam-free exposed area (r = −0.545; *p* < 0.001), reflecting the divergent development of the two variables during fermentation.

Separate analysis of each batch revealed considerable heterogeneity in the relationships among variables. For the pH–temperature association, the mean Pearson coefficient calculated across the 12 batches was r = −0.009 ± 0.277, and eight of the 12 batches had coefficients that were statistically significant at α = 0.05. The combination of an overall mean close to zero and significant coefficients in many batches indicates differences in direction and magnitude among fermentations and shows that an aggregate correlation may obscure batch-specific relationships.

Figure 4 presents the key relationships identified in the correlation analysis. Figure 4a shows the negative relationship between fermentation time and foam height (r = −0.686; *p* < 0.001), with a decrease from maximum values of ~60–70 mm during the first 20 h to below 10 mm after 80 h.

The positive correlation between foam-free exposed area and time (r = 0.876; *p* < 0.001), shown in Figure 4c, reflects the progressive exposure of the liquid surface as the foam collapsed. Figure 4d shows the decrease in pH over time (r = −0.574; *p* < 0.001). Stratification by extract concentration (12 °P, 15 °P and 18 °P) showed similar patterns among concentrations, with differences mainly in amplitude. To assess the robustness of these associations to within-batch temporal dependence, the correlations between time and foam-free exposed area, time and foam height, and time and pH were recalculated using data aggregated for the 11 batches whose fermentation-time column was logged rather than reconstructed (the mean of each variable per batch). At this level of aggregation, which reflects the experimental unit rather than the individual hourly frame, the associations were much weaker and not statistically significant: time–foam-free exposed area r = 0.359 (*p* = 0.278), time–foam height r = −0.409 (*p* = 0.212) and time–pH r = −0.412 (*p* = 0.208). This discrepancy indicates that the high values reported for the 1147 hourly observations largely reflect temporal pseudoreplication (autocorrelation among successive observations from the same batch) and should be interpreted as exploratory observation-level associations rather than as independent inferential evidence at the level of the experimental unit (the fermentation batch).

#### 3.2.4. Effect of Initial Wort Concentration on Foam Characteristics

Analysis by wort extract concentration level used batch means to reduce temporal dependence among observations. The temporal dynamics of foam height at each initial extract level (12 °P, 15 °P and 18 °P) are shown in Figure 5a–c, illustrating within-group variability (individual curves) and the mean trend (Mean ± SEM) during fermentation.

Differences among wort extract concentration levels (°P) did not reach statistical significance for any variable tested at batch level (Table 3): temperature (H = 0.49; *p* = 0.782), pH (H = 3.26; *p* = 0.196), pO_2_ (H = 4.81; *p* = 0.090), foam height (H = 3.96; *p* = 0.138) and foam-free exposed area (H = 1.47; *p* = 0.479). Consistent with this finding, Figure 5 shows comparable kinetic profiles at 12 °P (*n* = 5), 15 °P (*n* = 4) and 18 °P (*n* = 3), with an early peak followed by a decrease. Differences among levels concerned amplitude and dispersion rather than a fundamentally different temporal pattern. The available data did not provide robust evidence of differences among extract levels for any of the variables tested; interpretation is limited by the small and unbalanced number of independent batches (5, 4 and 3 at 12, 15 and 18 °P), and a genuine effect of initial extract on foam characteristics can be neither established nor excluded from this design.

#### 3.2.5. Temporal Development of Physicochemical Parameters

Linear-regression analysis of temporal development identified consistent trends in the principal variables. The pH decreased in 11 of the 12 fermentations (slopes from −0.003 to −0.010 pH units/hour; R^2^ = 0.116–0.656; *p* < 0.05). The mean change in pH from the first to the final measurement was −0.75 ± 0.16 units.

pO_2_ showed decreasing trends over time, with slopes from −0.002 to −0.088%/hour (R^2^ = 0.003–0.696). The change in pO_2_ between the first and last measurements was −2.50 ± 4.50% (*n* = 12 batches).

Temperature showed mixed trends among the fermentations (five positive slopes and seven negative slopes), ranging from −0.039 to 0.022 °C/hour (R^2^ = 0.001–0.734). The difference between the first and final measurements was 0.04 ± 2.17 °C (range: −6.20 to 3.09 °C; *n* = 12 batches). These mixed trends are compatible with contributions from fermentation exothermy and from the dynamics of the temperature-control loop, although neither contribution was quantified separately in this study.

#### 3.2.6. Characterisation of Fermentation Maxima (Endpoints)

Peak foam height ranged from 36.73 to 69.20 mm, with a mean of 53.52 ± 12.20 mm (*n* = 12 batches). The time to peak foam height was 16.34 ± 5.34 h (range: 6.00–26.79 h; *n* = 12 batches). The observed peak therefore occurred during the early active-fermentation period [38].

Maximum foam-free exposed area ranged from 6775.28 to 13,718.26 mm^2^, with a mean of 9999.81 ± 2597.81 mm^2^ (*n* = 12 batches). The time to maximum foam-free exposed area was 89.83 ± 16.69 h (range: 62.00–117.00 h; *n* = 12 batches), indicating that maximum exposure of the foam-free surface occurred during the late stages of fermentation as the foam layer diminished and more of the liquid surface became exposed.

Areas under the curve (AUCs) for time-dependent variables were calculated to characterise cumulative process behaviour throughout fermentation. The AUC integrates both response magnitude and duration, allowing batches to be compared beyond individual or maximum observed values. For foam height, the AUC ranged from 1184.49 to 2445.95 mm·h, with a mean of 1759.17 ± 442.28 mm·h (*n* = 12 batches). In this context, a higher AUC indicates more intense or more persistent foaming, or a combination of both. For foam-free exposed area, the AUC was 522,888.20 ± 216,638.76 mm^2^·h, with values ranging from 266,975.83 to 975,189.88 mm^2^·h. This metric expresses the cumulative load of the surface classified as foam-free during fermentation by integrating its spatial extent and temporal persistence. AUC values therefore provide global indicators of fermentation dynamics that complement maximum values and observations from individual operational time windows.

Analysis of peak characteristics by extract concentration (°P) in Figure 6 showed different trends among variables, although none reached statistical significance with the available sample sizes (3–5 batches per level). Maximum foam height (panel a) was greatest at 18 °P (65.9 ± 3.7 mm), compared with 12 °P (50.7 ± 11.8 mm) and 15 °P (47.8 ± 11.9 mm), without a monotonic increase across the three levels (Kruskal–Wallis, *p* = 0.138). Maximum foam-free exposed area (panel c) showed no clear pattern by extract concentration; within-group variability (SD up to 3.2 × 10^3^ mm^2^) was comparable to differences among means (*p* = 0.576). Time to peak foam height (panel d) ranged from 6 to 27 h, with means of 18.4 ± 4.8 h (12 °P), 12.8 ± 6.8 h (15 °P), and 17.8 ± 4.1 h (18 °P). No systematic differences were observed among levels (H = 1.34; *p* = 0.511), suggesting that the kinetics of reaching the peak were less sensitive to substrate concentration than the peak amplitude. Given the small number of batches at each level, these results should be interpreted as trends; the absence of statistical significance should be interpreted cautiously given the limited statistical power and does not by itself indicate either the presence or absence of an effect.

### 3.3. Batch-Aware Mixed-Effects Sensitivity Analysis

In the sensitivity analysis based on the 70 batch-by-window means from 12 batches, operational time window was associated with foam height (F(5, 53.10) = 72.17; *p* < 0.001) and with foam-free exposed area (F(5, 62.00) = 80.57; *p* < 0.001). Initial extract level was not statistically significant for foam height (F(2, 8.96) = 0.71; *p* = 0.520) or for foam-free exposed area (F(2, 62.00) = 0.92; *p* = 0.404). For foam height, the between-batch random intercept was estimated at SD = 0.207 against a residual SD of 0.323, so batch identity accounted for an appreciable share of the variation left once the time window was accounted for. For foam-free exposed area, the random-intercept variance was estimated at zero; that model therefore reduces to ordinary least squares, and its denominator degrees of freedom equal the residual degrees of freedom of the fixed-effect design. This is reported rather than concealed because it means the exposed-area data carry no detectable batch-level clustering beyond the time window. Given the small and unbalanced number of batches, the non-significant extract-level results indicate that an effect was not detected; they do not demonstrate the absence of an effect. Full model output is given in Table S4.

## 4. Discussion

### 4.1. Scope and Limits of the Computer-Vision Component

A factual clarification regarding the computer-vision component is important. The architecture recovered from the archived training implementation is a convolutional encoder–decoder network without encoder-to-decoder skip connections and without a pre-trained encoder; it therefore should not be interpreted as a standard U-Net implementation. U-Net terminology used in the original submission was consequently removed from the title, keywords and claims of architectural performance during the previous revision. The present study makes no claim of architectural novelty.

It must nevertheless be acknowledged that the retained segmentation component has important technical weaknesses, particularly under sparse-foam conditions. These weaknesses are visible in the archived same-batch validation results and are reported rather than concealed. The original development procedure used separate per-fermentation models, only three to five same-batch validation frames per model, no independent test subset, and a random within-batch partition without a fixed seed, so the archived validation figures cannot by themselves support a controlled comparison. Because the annotated frames and their ground-truth masks were retained for all twelve fermentations, however, it was possible to construct such a comparison directly rather than infer it from the archive.

A fixed partition was therefore defined at the level of complete fermentations, and the baseline configuration was retrained from scratch alongside the modified configurations under identical conditions, so that any difference is attributable to the loss function or to the architecture rather than to partition variability (Section 2.5.3). The results are reported in Section 4.4 and in Supplementary Table S7. This experiment does not alter the foam descriptors analysed in Section 3, which were derived from the original per-fermentation models; it addresses the separate question of whether the known weakness of the segmentation component on sparse foam can be reduced.

The findings support the proof-of-concept feasibility of the complete workflow—longitudinal image acquisition, convolutional semantic segmentation, descriptor extraction and batch-aware statistical analysis—for descriptive monitoring of fermentation foam under the experimental conditions of this study; they do not establish the robustness of the segmentation step itself as a metrological instrument. For the per-fermentation models that generated the descriptors reported here, segmentation accuracy was not established on data independent of model development: a separate model was trained for each fermentation on that fermentation’s own annotated frames and validated on the remainder of the same frames. The targeted experiment of Section 2.5.3 provides limited independent-batch evidence for a separately trained pooled model on three withheld fermentations, but it does not retrospectively validate the descriptor-generating models, whose accuracy on unseen fermentations therefore remains unknown. Because consecutive frames of the same fermentation are visually highly similar, such a partition cannot exclude optimistic estimates, and the class-wise overlap actually recorded for these models (Section 3.1) was low and highly variable. U-Net remains a foundational architecture for semantic segmentation in biomedical and industrial applications because its encoder–decoder structure and skip connections recover spatial detail [20]; the network implemented here had an encoder–decoder structure but neither skip connections nor a pre-trained encoder, which is one plausible contributor to the weak class-wise overlap recorded in Section 3.1 and which motivates the architectural directions set out in Section 4.4.

Published work on the computational monitoring of bioprocesses provides context for the present study but not a basis for direct quantitative comparison. Hoseini, Rundquist, Poux and Aubin [26] assessed U-Net models, including attention-gated and residual variants, for the detection and automated quantification of foam in a stirred tank, and a Dice coefficient of 0.86 has been reported for segmenting foam texture in wastewater-treatment plants under both centralised and federated training [6]. Neither result can be set against the values recorded here. The archived evaluation of the present per-batch models reports intersection-over-union rather than Dice, and it was computed on validation frames drawn from the same fermentation as the training frames, whereas the cited studies report Dice under different validation schemes; the two sets of numbers are therefore not commensurable, and no equivalence of performance is claimed. Jeong and Kim [18] classified airlock images by the presence or absence of gas bubbles and aggregated those classifications into a cumulative fermentation index; that is a whole-image classification task rather than the pixel-level segmentation used here, so its output is not commensurable with the class-wise overlap reported in Section 3.1. Systems based on indirect image measurements, such as RoboBEER, report correlation coefficients of about 0.9 with human sensory panel scores [11] but rely on aggregated frame characteristics rather than pixel-level segmentation. Separately, 3D-camera approaches have proved more robust than standard RGB imaging for objective measurement of foam stability under real conditions, where light reflections and foam adhesion to vessel walls constrain methods based solely on contour detection [10].

Establishing generalisation beyond the visual characteristics of the batches used for training requires a partition at the level of complete fermentation batches, together with an independent test subset. Such a partition was not used for the per-fermentation models that generated the reported descriptors, so their generalisation to unseen batches remains untested. It was used for the targeted experiment of Section 2.5.3, which provides limited evidence on three withheld fermentations for a separately trained pooled model; extending that design to the descriptor-generating models remains the principal methodological priority for further work.

No uncertainty quantification of the kind recommended by García Fernández, et al. [39] for artificial neural networks was possible for the present configuration. Small evaluation sets are known to produce very wide confidence intervals [40]. The validation subsets available here held only three to five frames per fermentation, so no interval estimate of segmentation accuracy would have been informative; any future estimate will require an evaluation subset substantially larger than this, drawn from fermentations that contributed no training frames.

Difficult visual conditions such as specular reflections on the curved glass wall, condensation or residue, foam adhering above the bulk interface, fragmentation of the foam boundary and partial occlusion by sensors or the impeller are expected to produce large frame-level errors in foam-height estimation; their frequency was not quantified in this study. A Dice coefficient of about 0.86 has been reported as acceptable performance in published foam-segmentation work in bioprocesses [6,26]. Because the archived evaluation of the per-batch models used here reports intersection-over-union rather than Dice (Section 3.1), and does so on same-batch validation frames, a direct numerical comparison with those studies is inappropriate. The recorded intersection-over-union values were nevertheless low and highly variable and do not support claims of measurement equivalence or of generalisation to unseen fermentations. Claims of comparability with manual measurements are therefore not supported by the present evidence, and the descriptors should be read as relative, within-batch indicators rather than as calibrated absolute measurements.

No inference-time measurement is archived for the present configuration, so no claim is made about computational throughput. Accuracy–efficiency trade-offs have been documented for other lightweight architectures used in industrial segmentation [41].

### 4.2. Foam Dynamics in Relation to Operational Time Window and Wort Extract Level

The Friedman test identified a strong, consistent pattern of between-batch variation associated with the fermentation time window. The two image-derived descriptors showed strong concordance across operational time windows (Kendall’s coefficient of concordance W = 0.898 for foam height and W = 1.000 for foam-free exposed area); across all four variables with a significant window effect, W ranged from 0.470 to 1.000. By contrast, no robust differences among wort extract concentration levels (°P) remained after correction for multiple Kruskal–Wallis tests. This finding should be interpreted strictly as strong agreement in the pattern of between-batch variation across time windows—not as a proportion of variance explained or as a causal relationship. At the descriptive level, however, it indicates that foam morphology follows a pattern more associated with the temporal sequence of fermentation than with initial substrate concentration.

The temporal profile followed a consistent pattern: an increase to a maximum during High Kräusen (height 43.37 ± 12.80 mm), followed by a progressive decline to values comparable with the Lag phase during the Final phase. One plausible explanation is a change in CO_2_-production kinetics, which would be expected to peak during maximum yeast metabolic activity and to decrease as fermentable substrate is depleted. Neither the CO_2_ evolution rate nor any interfacial property was measured in this study, so this remains a hypothesis rather than a demonstrated mechanism. It is also consistent with reports that foam height increases with sustained bubble nucleation and accumulation of stabilising mannoproteins [42,43]. The low foam-descriptor values in the Lag-labelled window, and their rise through the Growth and High Kräusen windows, are compatible with the limited CO_2_ production expected during early metabolic adaptation and with its subsequent increase; the monotonic pH decline is likewise compatible with glycolytic production of organic acids and net proton release during amino-acid assimilation. Neither CO_2_ evolution nor organic-acid concentrations were measured, and the windows are fixed time intervals rather than physiological transitions confirmed for each batch, so these readings are interpretations and not results.

An association between initial wort concentration and foam morphology was not detected. Neither foam height nor foam-free exposed area differed significantly among the three wort extract concentration levels (°P) at batch level, and only 3–5 batches were available per level; the batch-aware mixed-effects analysis likewise did not detect an effect of extract level (*p* = 0.520 and *p* = 0.404). Given the small and unbalanced sample, this does not demonstrate the absence of an effect. Physicochemical mechanisms of foam formation and stabilisation may nevertheless contribute to between-batch differences in foam morphology, although the present design does not permit causal inference. The concentration of surfactants, including proteins and biosurfactants, is a recognised determinant of bubble size and foam stability; below the critical micelle concentration, increasing surfactant concentration reduces bubble size [44,45]. At 18 °P, wort may contain a higher concentration of surface-active constituents expected at higher extract levels, such as hydrophobic proteins and biosurfactants, which could reduce surface tension and help stabilise the gas–liquid interface; these compounds were not directly quantified in this study. The 18 °P batches had the greatest batch-level mean foam height (22.49 ± 2.25 mm), compared with 12 °P (17.33 ± 4.04 mm) and 15 °P (15.93 ± 4.06 mm), although this difference did not reach statistical significance.

Several physicochemical mechanisms could in principle account for a shift in foam morphology over the course of a fermentation. They are set out here as hypotheses rather than findings because none of the quantities involved was measured in this study. The superficial velocity of the carbon dioxide leaving the wort sets the rate at which gas is delivered to the foam layer, and therefore the time available for bubbles to coalesce before the films between them drain; a higher gas flux shortens that time and tends to sustain a finer, taller foam, whereas the declining flux of the late windows leaves the existing lamellae exposed to coalescence for longer. Coalescence itself is governed by the drainage of the thin liquid films separating adjacent bubbles and by whether the approach velocity of the interfaces exceeds the critical value for film rupture [46]. Both processes are modulated by an interfacial composition that does not stay constant: as fermentation proceeds, ethanol accumulates, pH falls by about 0.75 units, and yeast releases mannoproteins and hydrophobic polypeptides, each of which alters the dynamic surface tension of the wort and the viscoelasticity of the adsorbed interfacial layer [47]. Foam-positive malt polypeptides and yeast mannoproteins adsorb at the gas–liquid interface and confer steric and viscoelastic stabilisation on the films [48], while the surfactant concentration relative to the critical micelle concentration governs the bubble size produced at nucleation [44,45]. Testing any of these explanations would require direct measurement of the CO_2_ evolution rate, dynamic surface tension, interfacial rheology and wort protein composition, none of which was undertaken here.

### 4.3. Relationships Between Image-Derived Morphological Descriptors and Conventional Process Variables

Correlation analysis of the 1147 hourly observations identified associations between temporal dynamics and visual indicators of foam: fermentation time was negatively related to foam height, positively related to foam-free exposed area, and negatively related to pH. These observation-level correlations are nevertheless susceptible to temporal pseudoreplication. The 1147 observations are not independent experimental units but comprise 12 correlated time series. When the same associations were recalculated using data aggregated for the 11 batches whose fermentation-time column was logged rather than reconstructed—aggregation to the level of the experimental unit, restricted here to the batches carrying a directly logged time axis—the coefficients decreased substantially, and all three associations became statistically non-significant (*p* > 0.2). This discrepancy does not invalidate the descriptive value of observation-level correlations for characterising within-batch temporal patterns, but it requires them to be interpreted as exploratory associations rather than as robust experiment-level inferential evidence.

The pH remains a useful surrogate indicator of fermentation progress, with a consistent monotonic change during the process (a decrease of 0.75 units, from 4.69 in Lag to 3.94 in Final), although this does not independently validate it as a universal process marker. By contrast, pO_2_ and temperature showed no robust linear associations with foam height in the bivariate analysis, suggesting that their effects on foam morphology are either indirect or mediated by other process factors. Neural-network-based soft sensors can infer biological variables from standard physicochemical signals and could help address this indirect relationship in future work [49,50].

### 4.4. Limitations, Generalisability, and Industrial Translation

The number of batches was small (*n* = 12) and unevenly distributed among wort concentrations (5/4/3 at 12/15/18 °P), limiting statistical power for small effects, particularly at 18 °P (*n* = 3). No a priori formal power analysis was performed, consistent with the exploratory design. Temporal pseudoreplication arising from repeated hourly measurements within the same batch, discussed in Section 4.3, remains the most important methodological limitation of the statistical inferences reported for the 1147 observations. Pixel-level annotation covered 223 of the 1147 frames (19.4%) across all 12 batches and was confined to the first 72 h. Because the Final window begins at 55 h, 198 of the 505 observations in that window fall within the annotated temporal span and 307 do not; those 307 observations, 26.8% of the whole dataset, lie beyond the latest annotated frame. All morphological values reported beyond 72 h, including foam collapse and maximum exposed area, should therefore be interpreted as exploratory image-derived measurements from an unvalidated late-stage temporal domain. In addition, each network was trained and validated on frames from the single fermentation to which it was then applied, with no independent test subset, so the reported descriptors carry no accuracy figure established on data independent of model development; the independent-batch figures reported in Section 4.4 apply to a separately trained pooled model and not to these networks.

The recorded temperatures departed from the nominal 20 °C setpoint. Across the 11 batches excluding the batch containing the two isolated extremes, observed batch ranges extended from 19.20 to 25.32 °C; in the remaining batch, isolated readings of 27.5 °C and 11.5 °C occurred adjacent to values of approximately 21.6–21.8 °C. Without an independent reference logger, these readings cannot be attributed definitively to sensor or acquisition error rather than to short-term process fluctuation, so temperature is treated as an uncontrolled potential confounder rather than as evidence of effective thermal control.

Evaluation was performed exclusively in a small-volume bioreactor (2 L) with standardised geometry, a single yeast strain (SafAle™ S-33) and a single wort recipe. Generalisation to other geometries, strains or wort compositions was not quantified and requires external validation using batches entirely independent of those used for model development and evaluation. The computer-vision system remains sensitive to variation in illumination and to image-quality degradation during prolonged fermentation (condensation and residue on vessel walls), limitations similar to those reported in other foam-detection applications [26]. Because initial wort extract was the only experimentally manipulated factor and all remaining variation was observed rather than controlled, the design does not permit firm causal inferences; the identified correlations between process variables and foam characteristics do not demonstrate direct causality.

The economic impact of industrial implementation cannot be quantified rigorously without a dedicated cost–benefit analysis, and laboratory performance will not be reproduced identically in industrial installations without adapted calibration and validation protocols. Priorities for future research include expanding the dataset with additional batches balanced across wort extract concentration levels (°P) and different yeast strains; defining a fixed partition at the level of complete fermentation batches with an independent test subset and quantifying segmentation accuracy on it; externally validating the segmentation model on entirely independent fermentations; modelling temporal dependence explicitly, for example using mixed models with a random batch effect or generalised additive mixed models, to replace raw correlations as the principal inferential test; and integrating multimodal sensors, such as NIR spectroscopy and gas analysis, to complement the limitations of each measurement modality considered separately [51].

Class imbalance in this dataset is a property of individual frames rather than of the collection as a whole. Foam accounts for 26.7% of all annotated pixels, but the per-frame distribution is strongly skewed: 28.3% of frames contain less than 5% foam pixels and 15.2% contain less than 0.5%, and every one of the twelve fermentations contributes such frames. These sparse-foam states occur in the Lag and Final windows and correspond to the failure mode catalogued in Table S6 and to the fermentation in which the foam class was not recovered at all (Table S5). A targeted experiment was therefore carried out to establish whether this weakness can be reduced (Section 2.5.3), using attention gating as implemented in Attention U-Net [52] and the asymmetric Tversky loss [53]. Boundary-aware alternatives such as boundary loss [54] were not tested and remain candidates for further work.

Replacing class-weighted cross-entropy with the Tversky loss, while leaving the archived encoder–decoder architecture and every training setting unchanged, increased per-frame foam intersection-over-union on the three withheld fermentations by 0.043 (95% confidence interval 0.016 to 0.069; Wilcoxon signed-rank *p* = 0.005; r = 0.39). The improvement was concentrated where it matters: on the eight test frames containing less than 5% foam pixels, foam recall rose from 0.074 to 0.318 and intersection-over-union from 0.043 to 0.083, and the frame in which the baseline recovered no foam at all was recovered. Both Tversky configurations exceeded the baseline in each of the three withheld fermentations considered separately (Table S7), so the direction of the effect does not depend on a single test batch. Because the 52 paired frames derive from only three independent fermentations, this comparison is a paired frame-level analysis conditional on those batches rather than inference over independent experimental units, and it is reported as technical support rather than as a batch-level inferential test.

The attention-gated configuration behaved differently. Under weighted cross-entropy, it did not improve foam intersection-over-union relative to the encoder–decoder (0.001; *p* = 0.87), while it raised mean intersection-over-union across the six classes from 0.205 to 0.309, almost entirely through the background class (0.001 to 0.549). Because the skip connections and the attention gates were introduced together, this multi-class gain cannot be attributed to the attention mechanism specifically; restoration of spatial detail by the skip connections is at least as plausible an explanation. Combining attention gating with the Tversky loss gave the highest aggregate foam intersection-over-union (0.524; 0.054 above baseline; *p* < 0.001; r = 0.73), yet it failed to recover foam in four of the eight sparse-foam frames, against none for the Tversky loss on the encoder–decoder.

No single configuration is therefore uniformly preferable, and the asymmetric loss carries a measurable cost: it reduced mean intersection-over-union across the six classes from 0.205 to 0.178 and markedly degraded the metal class, which is the expected consequence of weighting false negatives more heavily than false positives. For monitoring applications in which thin foam layers must be detected, the encoder–decoder with Tversky loss is the more appropriate configuration; for balanced multi-class segmentation, the tested architecture combining skip connections and attention gates performed better, although the present design cannot attribute that advantage to the gating mechanism specifically.

These findings rest on a single partition and a single random seed, the sparse-foam comparison rests on eight frames, and training used 256 × 256 inputs for computational feasibility, so the magnitudes should be regarded as indicative rather than as stable estimates of effect. The experiment also trains one pooled model, whereas the descriptors analysed in Section 3 come from twelve per-fermentation models, so these values are not directly comparable with the archived per-batch evaluation of Table S5 and do not supersede it. They nevertheless establish that the class imbalance affecting the foam class is reducible by a loss function chosen for that purpose, and that the attention-gated configuration tested here did not improve it under weighted cross-entropy.

Overall, this study indicates that a computer-vision-based workflow for fermentation monitoring built on semantic segmentation is feasible as a proof of concept and can provide information complementary to conventional sensors at the at-line resolution used (Δt = 60 min). The findings should be interpreted cautiously in view of the small sample, temporal pseudoreplication and the need for extensive external validation before translation to industrial conditions.

## 5. Conclusions

This study implemented and applied a method for monitoring alcoholic fermentation based on convolutional semantic segmentation integrated with multiparametric sensor-data acquisition. Across 12 independent fermentations, image-derived estimates of foam height and foam-free exposed area were extracted from hourly images and analysed together with synchronised pH, temperature and dissolved-oxygen records. Because a separate segmentation model was trained for each fermentation and evaluated only on frames from that same fermentation, segmentation accuracy for those models was not established on independent data; a targeted experiment on a separately trained pooled model provides limited independent-batch evidence but does not validate them retrospectively. The study therefore demonstrates the proof-of-concept feasibility of the acquisition and analysis workflow rather than a validated measurement performance of the segmentation step.

Analysis of all experimental observations identified a morphological pattern strongly associated with operational time window (Kendall’s coefficient of concordance W = 0.898 for foam height and W = 1.000 for foam-free exposed area), while initial wort concentration had no robust association with the tested morphological variables among the available independent batches after correction for multiple testing. These findings support further investigation of window-aligned monitoring, which requires confirmation in a larger and balanced set of batches before any control application.

Correlations between foam characteristics and physicochemical process variables were strong in magnitude at the level of individual hourly observations but weakened substantially and became non-significant after batch-level aggregation. They should therefore be interpreted as exploratory associations rather than as independent experiment-level inferential evidence. In the batch-aware mixed-effects sensitivity analysis, operational time window was associated with both foam height and foam-free exposed area (both *p* < 0.001), whereas an effect of initial extract level was not detected (*p* = 0.520 and *p* = 0.404). In this context, pH remains a consistent monotonic indicator of fermentation progress.

From an applied perspective, the findings indicate that image-based descriptors can characterise foam dynamics at a resolution useful for laboratory process monitoring. They do not establish suitability for quantitative process control or for identifying physiological phases. Their interpretation must nevertheless acknowledge the methodological limitations that constrain the validity and generalisability of the conclusions: the small and unbalanced sample (*n* = 12 batches; 5/4/3 across wort extract concentration levels, °P); pseudoreplication from repeated hourly measurements within each batch; validation limited to one bioreactor type, one yeast strain and one wort recipe; and the absence of independent-batch validation of the descriptor-generating per-fermentation models. With initial wort extract as the only manipulated factor, the design does not permit firm causal inference.

Future research should prioritise expansion of the experimental dataset with additional balanced batches, extension of the fixed batch-level partition used in the targeted experiment of Section 2.5.3 to the descriptor-generating workflow with quantification of segmentation accuracy on it, external validation of the segmentation model, explicit modelling of temporal dependence—for example, through random batch effects—in place of raw correlations as the principal inferential test, and integration with complementary sensor modalities for multimodal characterisation of fermentation dynamics.

The contribution of this study should therefore be distinguished from architectural innovation in computer vision. The semantic-segmentation network functioned as an image-analysis component whose limitations are explicitly reported; the principal contribution is the longitudinal, multi-batch characterisation framework that links image-derived foam behaviour with synchronised fermentation-process measurements while accounting for the fermentation batch as the experimental unit. A targeted experiment under a fixed batch-level design showed that an asymmetric loss function reduces, without eliminating, the failure of the segmentation component on sparse-foam frames, whereas the tested configuration combining skip connections and attention gates did not. Establishing whether these differences persist across repeated partitions, and whether they translate into more accurate foam descriptors, remains necessary before any claim of deployment generalisation is made.

## Figures and Tables

**Figure 1:**
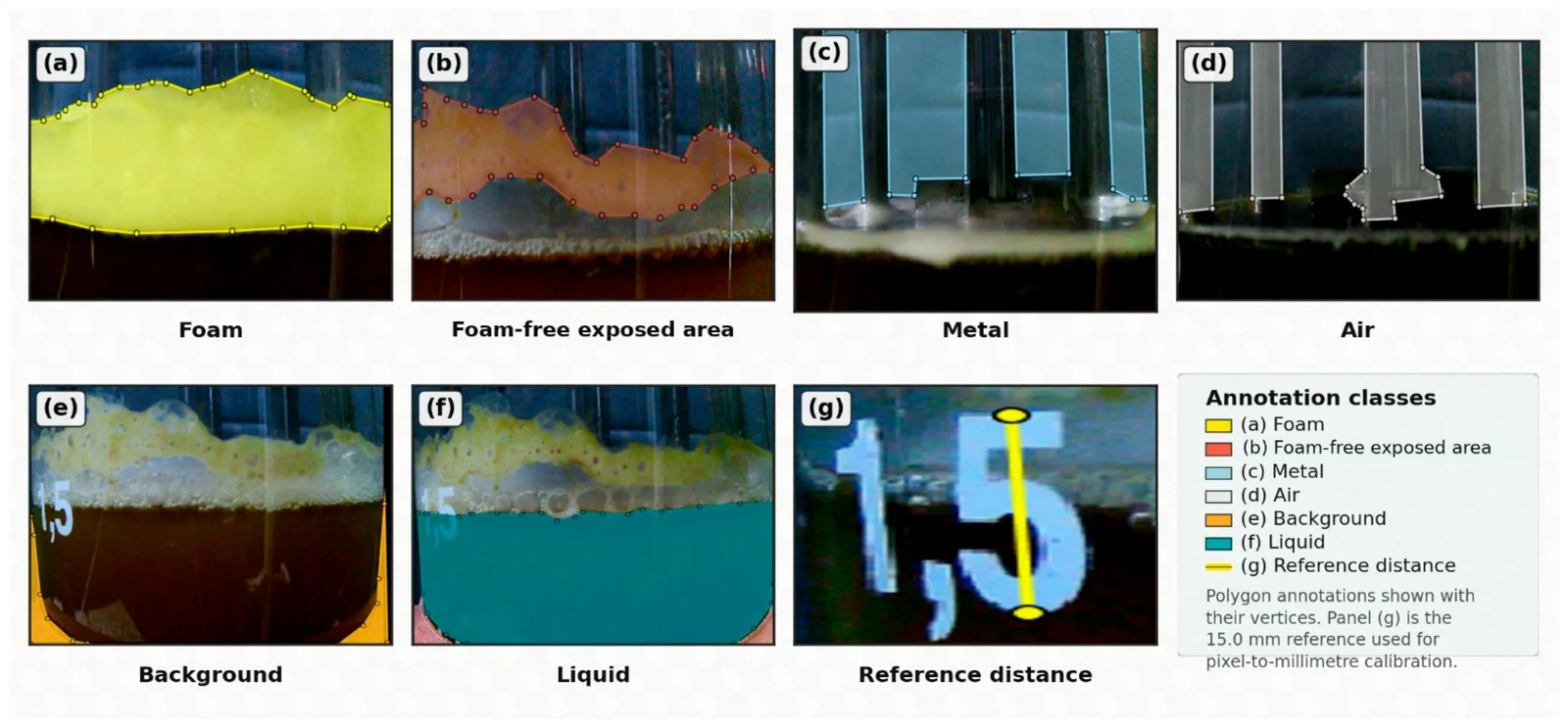
Label taxonomy used for pixel-level semantic annotation in the Image Labeler app (Computer Vision Toolbox version 24.1, MATLAB R2024a): (**a**) foam region—yellow polygon; (**b**) foam-free exposed area—brown polygon; (**c**) metal components—light-blue polygon; (**d**) residual gas spaces (air)—white polygon; (**e**) background elements—orange polygon; (**f**) liquid phase—turquoise polygon; (**g**) spatial calibration marker—yellow line (15.0 mm). The “1,5” visible in panels (**e**–**g**) is the factory-moulded 1.5 L volume graduation on the glass vessel and is not a data label; the yellow line in (**g**) spans a reference distance of 15.0 mm across this graduation.

**Figure 2:**
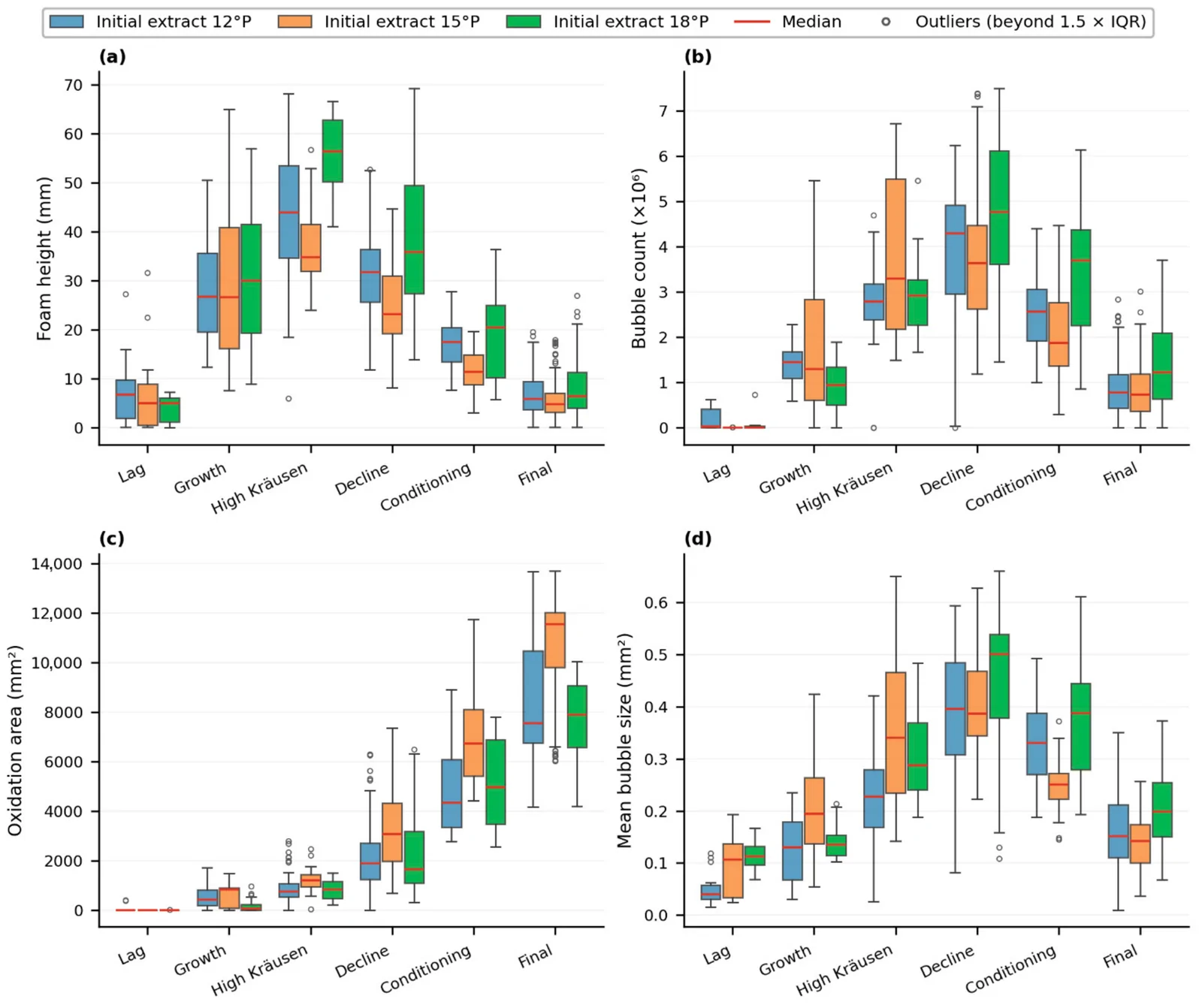
Foam characteristics by operational time window (Lag, Growth, High Kräusen, Decline, Conditioning and Final), stratified by initial extract level (12 °P, 15 °P and 18 °P). Distributions are shown for (**a**) the image-derived foam-height estimate (mm); (**b**) the image-derived bubble count (×10^6^); (**c**) the image-derived foam-free exposed-area estimate (mm^2^), labelled “Oxidation area” on the panel axis; and (**d**) the image-derived mean bubble size (mm^2^). The bubble-count and mean-bubble-size descriptors shown in (**b**,**d**) are reported descriptively and were not used in the analyses presented in this article. Box plots show the median (line), interquartile range (IQR; box), whiskers (1.5 × IQR) and extreme values (points).

**Figure 3:**
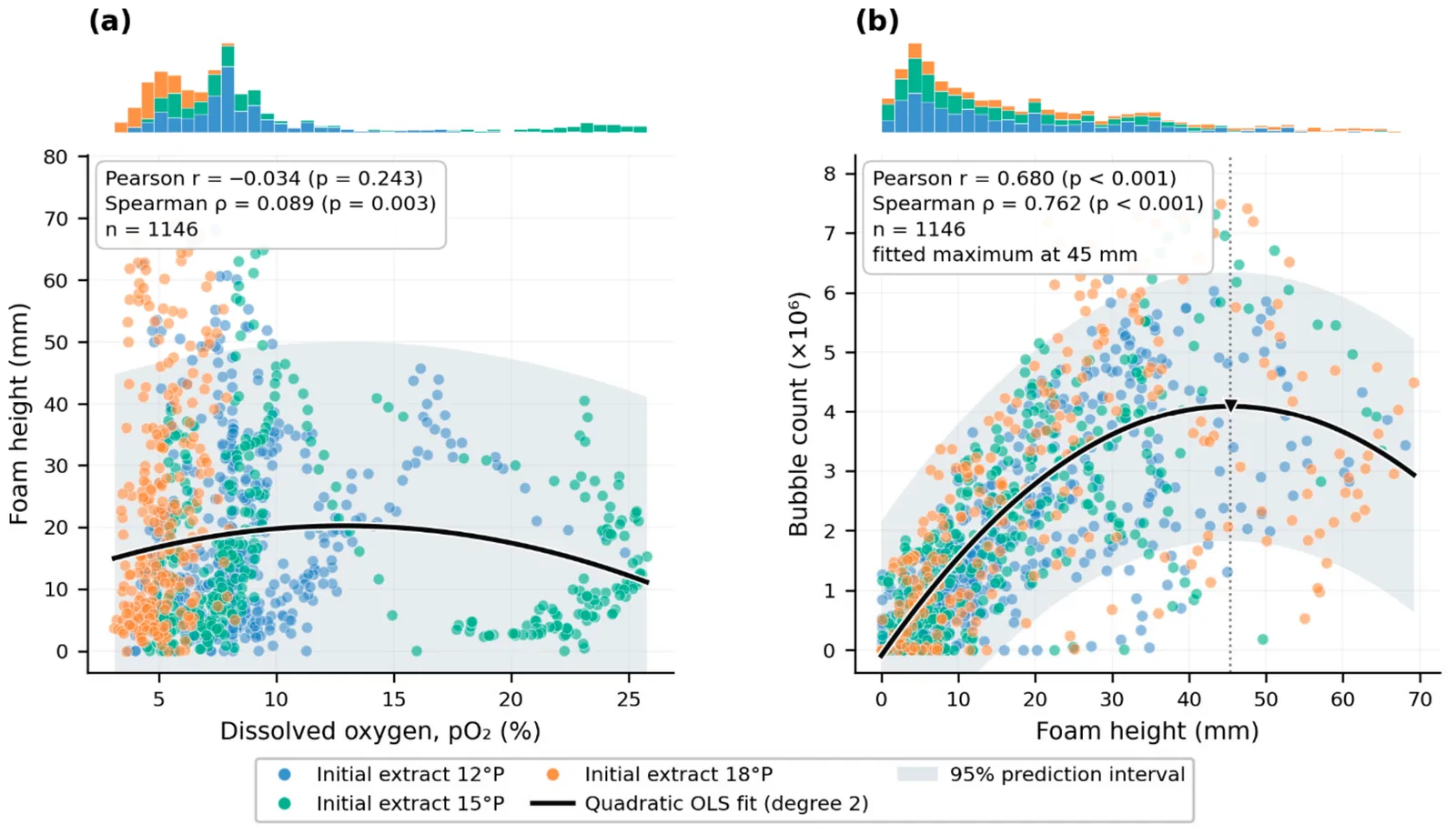
Image-derived foam-height estimate as a function of dissolved pO_2_ (**a**), and image-derived bubble count as a function of the image-derived foam-height estimate (**b**), with observations stratified by initial extract concentration (12 °P, blue; 15 °P, green; 18 °P, orange). The shaded band is the 95% prediction interval of the quadratic ordinary-least-squares fit; in panel (**b**) the vertical dotted line and the triangle mark the position of the fitted maximum, at a foam height of 45 mm. The bubble-count descriptor in (**b**) is shown descriptively and was not used in the analyses presented in this article. In panel (**a**) the linear association was negligible and not statistically significant (Pearson r = −0.034; *p* = 0.245), while the monotonic association was very weak (Spearman ρ = 0.090; *p* = 0.002). Values are based on 1147 hourly observations nested within 12 fermentation batches and are exploratory at the observation level.

**Figure 4:**
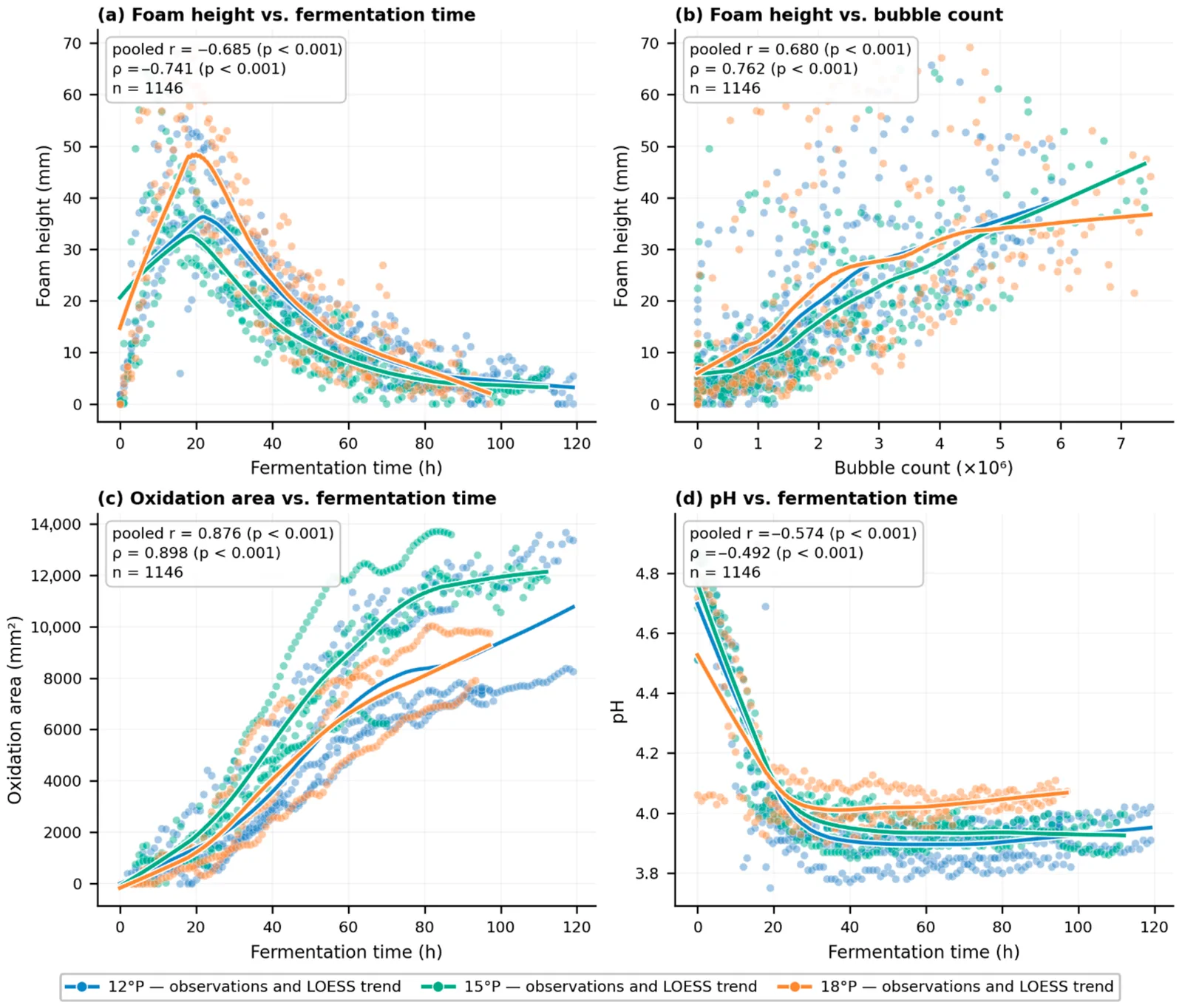
Relationships between process variables and foam characteristics (1147 hourly observations nested within 12 batches), stratified by initial extract level: 12 °P (blue), 15 °P (green) and 18 °P (orange). (**a**) Image-derived foam-height estimate vs. fermentation time; (**b**) image-derived foam-height estimate vs. image-derived bubble count; (**c**) image-derived foam-free exposed-area estimate vs. fermentation time, labelled “Oxidation area” on the panel axis; and (**d**) pH vs. fermentation time. The solid lines are LOESS trends fitted within each extract level. The bubble-count descriptor in (**b**) is shown descriptively and was not used in the analyses presented in this article. These observation-level relationships are exploratory.

**Figure 5:**
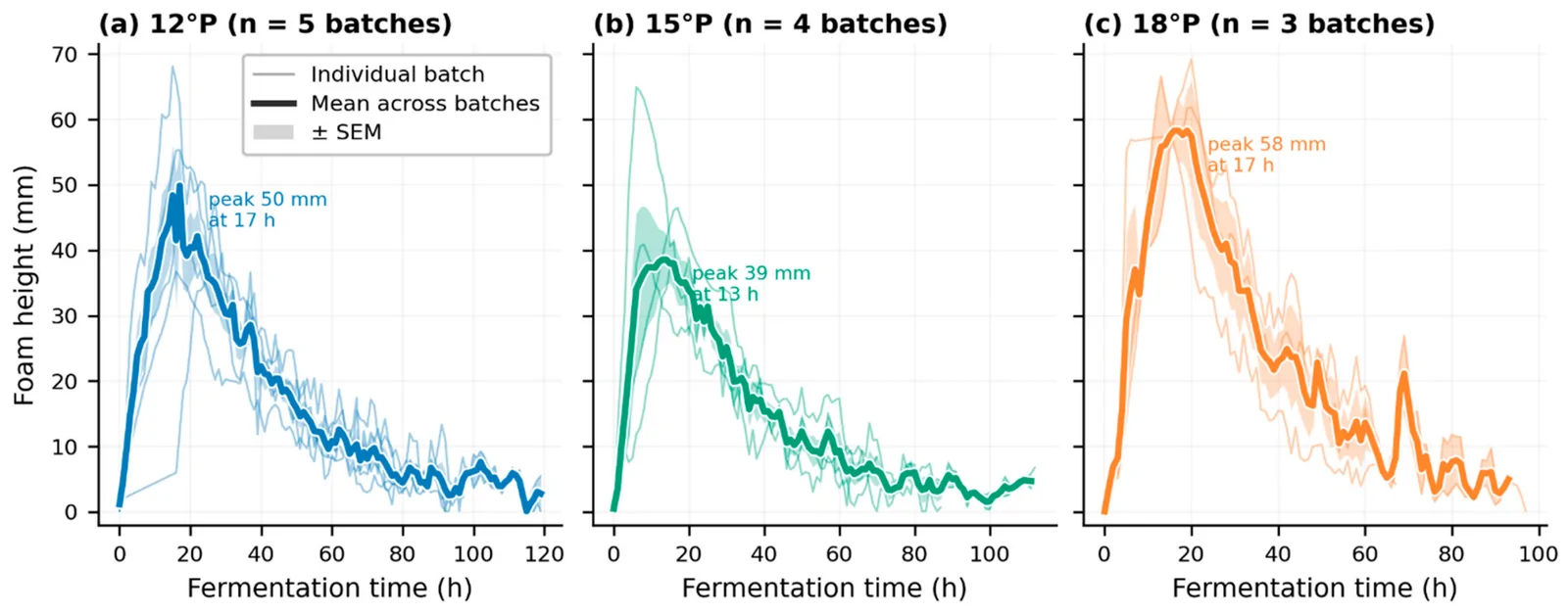
Dynamics of the image-derived foam-height estimate as a function of fermentation time at different wort extract concentration levels (°P). Profiles are shown for (**a**) 12 °P (*n* = 5), (**b**) 15 °P (*n* = 4) and (**c**) 18 °P (*n* = 3). Thin lines represent individual batches, and the thick line represents the Mean ± SEM.

**Figure 6:**
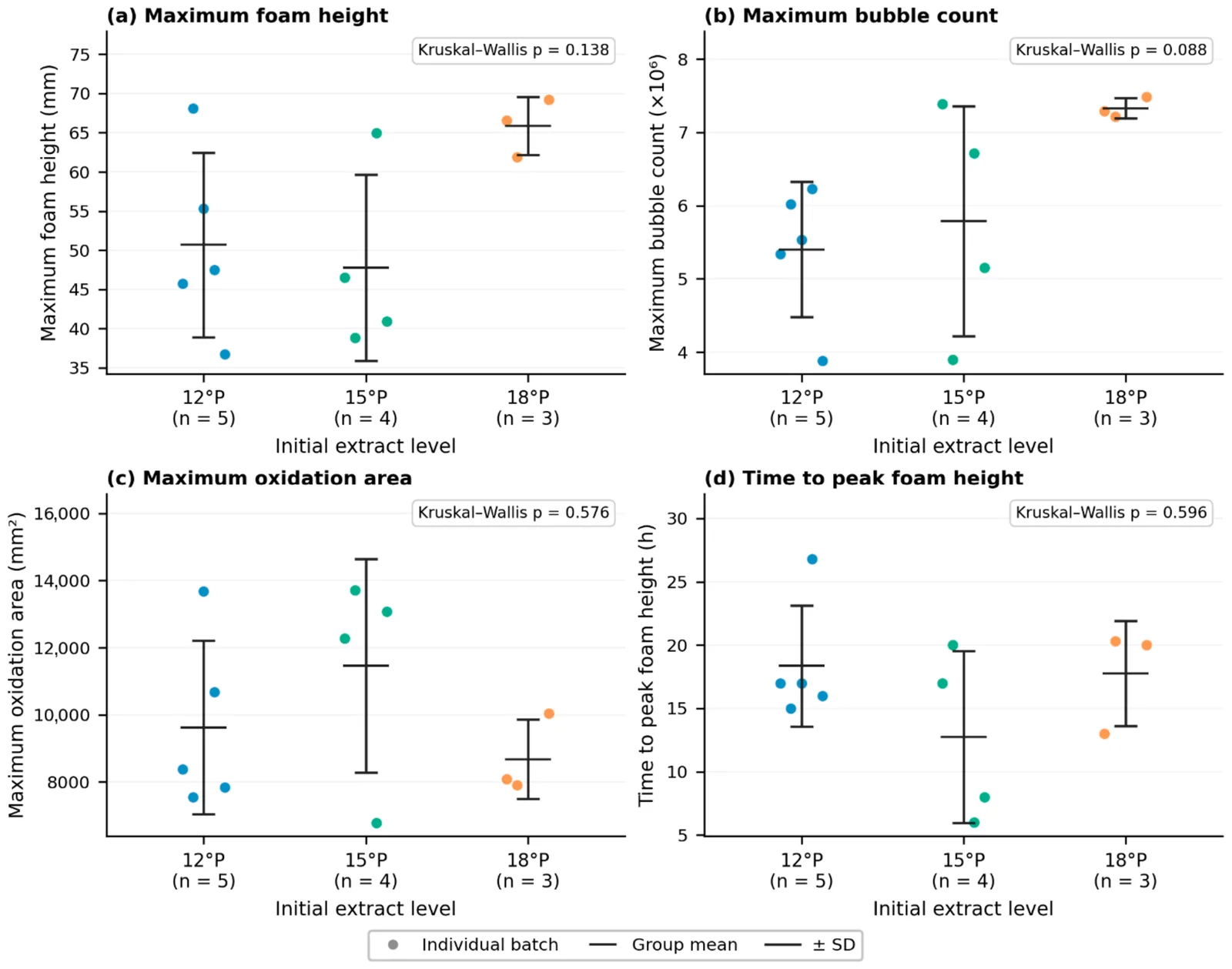
Peak fermentation characteristics by initial extract concentration (*n* = 12 batches; 12 °P: 5 batches, 15 °P: 4 and 18 °P: 3). (**a**) Maximum image-derived foam-height estimate (mm); (**b**) maximum image-derived bubble count (×10^6^); (**c**) maximum image-derived foam-free exposed-area estimate (mm^2^), labelled “Maximum oxidation area” on the panel axis; and (**d**) time to peak foam height (h). The bubble-count descriptor in (**b**) is shown descriptively and was not used in the analyses presented in this article. Each point represents one batch; the horizontal line indicates the group mean and the bars indicate ± standard deviation. Differences among levels were tested using the Kruskal–Wallis test.

**Table 1:** Descriptive statistics for fermentation variables: overall and window-specific values (*n* = 1147 observations).

Variable	Global (*n* = 1147)	Lag (*n* = 43)	Growth (*n* = 76)	High Kräusen (*n* = 81)	Decline (*n* = 263)	Conditioning (*n* = 179)	Final (*n* = 505)
Temperature (°C)	21.56 ± 1.24	21.79 ± 1.78	21.80 ±1.67	21.67 ±1.98	21.62 ±1.24	21.45± 1.12	21.49 ±0.98
pH	4.03 ± 0.22	4.69 ± 0.20	4.50 ± 0.18	4.17 ± 0.19	3.97 ± 0.08	3.94 ± 0.08	3.94 ± 0.07
pO_2_ (%)	8.88 ± 5.26	9.45 ± 5.64	10.29 ± 5.79	9.71 ± 5.06	9.27 ± 5.33	8.68 ± 5.40	8.36 ± 5.05
Foam height (mm)	17.84 ±15.25	6.28 ±7.04	30.40 ±15.26	43.37 ±12.80	31.15 ±11.68	15.85 ± 6.42	6.62 ±4.35
Foam-free exposed area (mm^2^)	5560.86 ± 3956.21	18.62 ± 83.06	478.80 ± 458.46	1040.54 ± 607.62	2553.24 ± 1610.36	5659.19 ± 2104.07	9054.13 ± 2415.89

Note: Values are presented as mean ± SD. Overall CV: Temperature 5.77%; pH 5.50%; pO_2_ 59.27%; Foam height 85.48%; Foam-free exposed area 71.14%. Overall range: Temperature 11.50–27.50 °C; pH 3.75–4.94; pO_2_ 3.10–25.76%; Foam height 0.00–69.20 mm; Foam-free exposed area 0.00–13,718.26 mm^2^. The Friedman test was applied to the batch means for each operational time window (*n* = 10 batches with complete window coverage). The 2 batches excluded from this test had not passed through all 6 operational time windows before monitoring ended.

**Table 2:** Between-batch variability in fermentation variables (*n* = 12 batches).

Variable	Overall Mean	Between-Batch SD	Between-Batch CV (%)
Temperature (°C)	21.62	1.18	5.44
pH	4.03	0.08	1.92
pO_2_ (%)	8.47	4.94	58.35
Foam height (mm)	18.15	4.31	23.75
Foam-free exposed area (mm^2^)	5439.12	1608.15	29.57

Note: SD = standard deviation; CV = coefficient of variation. The ‘overall’ mean in Table 1 is the weighted mean of the 1147 individual hourly observations; the ‘overall’ mean in Table 2 is the unweighted mean of the 12 batch means, with each batch contributing equally regardless of its number of observations. The two values are calculated differently and should not be compared directly.

**Table 3:** Comparison of variables by °P concentration.

Variable	12 °P	15 °P	18 °P	H	*p*
TEMP (°C)	21.67 ± 1.03	21.30 ± 1.24	21.95 ± 1.68	0.49	0.782
pH	3.99 ± 0.06	4.05 ± 0.08	4.08 ± 0.08	3.26	0.196
pO_2_ (%)	8.33 ± 2.45	11.23 ± 7.67	5.03 ± 0.98	4.81	0.090
Foam height (mm)	17.33 ± 4.04	15.93 ± 4.07	22.49 ± 2.25	3.96	0.138
Foam-free exposed area (mm^2^)	5324.83 ± 1755.80	6339.55 ± 1712.87	4429.01 ± 687.41	1.47	0.479

Note: Values are presented as mean ± SD (*n* = 5 for 12 °P; *n* = 4 for 15 °P; *n* = 3 for 18 °P). The reported *p* value is the nominal unadjusted value; no result remained statistically significant after Bonferroni correction for the five variables tested (corrected α = 0.05/5 = 0.0100).

## Data Availability

All quantitative results and evaluation metrics that support the conclusions of this study are reported in the article and in its Supplementary Materials; the results of the fixed batch-level test experiment on the three withheld fermentations are given in Table S7 and Figure S1. The underlying fermentation datasets, the annotated images and their ground-truth masks, the trained segmentation models, the training, inference and fixed-partition scripts, and the associated analysis scripts are not publicly available and cannot be released, because they are subject to ongoing institutional technology-transfer and commercialisation procedures and contain commercially confidential implementation information. For the duration of these procedures, no data, code or trained model beyond the material already reported in the article and its Supplementary Materials can be made available by the authors, including on individual request.

## Supplementary Materials

The following supporting information can be downloaded at: https://www.mdpi.com/article/10.3390/foods15193366/s1, Table S1: Exploratory observation-level Pearson correlations between process variables and foam characteristics; Table S2: Per-batch acquisition and pixel-level annotation inventory, with the partition of the annotated frames; Table S3: Properties of the partition used for per-batch model development; Table S4: Linear mixed-effects sensitivity analysis of the image-derived descriptors (70 batch-by-window cells from 12 fermentation batches); Table S5: Archived per-batch class-wise segmentation evaluation; Table S6: Catalogue of expected failure modes in image-based foam-height estimation; Table S7: Targeted class-imbalance experiment on a fixed batch-level partition; Figure S1: Targeted class-imbalance experiment on the three withheld fermentations.
