# Supplementary material for: Longitudinal Multi-Batch Characterisation of Beer Wort Fermentation Foam Using Image-Based Semantic Segmentation
Source: Foods. 2026 Sep 22;15(19):3366. doi: 10.3390/foods15193366 (PMC13664001; doi:10.3390/foods15193366)
Supplement: Supplementary file 1 [file foods-15-03366-s001.zip › foods-4547362-supplementary.pdf]

Supplementary Materials

Longitudinal Multi-Batch Characterisation of Beer Wort Fermentation Foam Using Image-Based Semantic Segmentation

Table S1. Exploratory observation-level Pearson correlations between process variables and foam characteristics.

Variable 1	Variable 2	r	p	n
Fermentation time	Foam-free exposed area (mm²)	0.876	< 0.001	1147
Fermentation time	Foam height (mm)	-0.686	< 0.001	1147
Foam height	Foam-free exposed area (mm²)	-0.676	< 0.001	1147
Fermentation time	pH	-0.574	< 0.001	1147
pH	Foam-free exposed area (mm²)	-0.545	< 0.001	1147

Note: r = Pearson correlation coefficient. Correlations involving fermentation time were calculated using the dataset with complete timestamps (n = 1147). Batch-level reassessment: to examine the robustness of correlations involving fermentation time to within-batch temporal dependence, the same three associations were recalculated using data aggregated for the 11 batches whose fermentation-time column was logged rather than reconstructed (the mean of each variable per batch). Results: Fermentation time–Foam-free exposed area r = 0.359 (p = 0.278; n = 11); Fermentation time–Foam height r = −0.409 (p = 0.212; n = 11); Fermentation time–pH r = −0.412 (p = 0.208; n = 11) — none was statistically significant at this level of aggregation. This batch-level reassessment applies only to the Pearson correlations involving fermentation time; the batch-by-window mixed-effects analysis reported in Table S4 includes all 12 fermentations. The values calculated for n = 1147 in the table above reflect associations at the level of individual hourly observations and should be interpreted as exploratory rather than as inferential tests at the level of the experimental unit (the fermentation batch). Correlations involving bubble count and mean bubble size have been removed: these variables are assigned in the archived analysis code by a step function of foam height rather than measured from the segmentation masks. Segmentation models were trained one per fermentation and validated on frames from the same fermentation, with no held-out test subset; the class-wise evaluation available for nine of these models is reported in Table S5.

Table S2. Per-batch acquisition and pixel-level annotation inventory, with the partition of the annotated frames.

**Fermentation**	**Initial extract (°P)**	**Observations acquired**	**Annotated frames**	**Training frames**	**Validation frames**	**Class-wise evaluation recorded**
13.03.2025	12	72	15	12	3	no
19.03.2025	15	111	20	16	4	yes
26.03.2025	15	71	14	11	3	yes
02.04.2025	18	98	20	16	4	yes
09.04.2025	12	104	19	15	4	yes
07.05.2025	15	113	23	18	5	yes
14.05.2025	18	84	22	18	4	yes
22.05.2025	12	120	24	19	5	no
05.06.2025	12	120	14	11	3	yes
10.07.2025	12	99	20	16	4	yes
22.07.2025	15	88	18	14	4	no
Subtotal — the 11 batches of the primary annotation inventory	5 / 4 / 2 batches at 12 / 15 / 18°P	1,080	209	166	43	8 of 11

01.07.2025 (outside the 209-frame primary inventory)	18	67	14	11	3	yes
Total — all 12 fermentations	5 / 4 / 3 batches at 12 / 15 / 18°P	1,147	223	177	46	9 of 12

Note: the number of observations acquired differs between fermentations (67 to 120) because acquisition ended at a different point in each run; the design is therefore unbalanced both in the number of batches per extract level (5 / 4 / 3) and in the number of hourly observations per batch. One network was trained per fermentation on that fermentation's own annotated frames; there was no pooled training set and no held-out test subset. Training and validation counts follow the partition rule implemented in the training script, which assigns round(0.8 × n) frames of a batch to training and the remainder of the same batch to validation. Annotation covered 223 frames across all 12 fermentations, distributed as 92 frames from 5 batches at 12°P, 75 frames from 4 batches at 15°P and 56 frames from 3 batches at 18°P; this is the figure quoted whenever the annotated dataset as a whole is described in the main text. Within the archive the 223 frames are held as two inventories: 209 frames from 11 fermentations, catalogued as the primary annotation inventory and distributed as 92, 75 and 42 frames at 12, 15 and 18°P, and the 14 frames of fermentation 01.07.2025, which were used only for its own model. The distinction is one of archival provenance and has no analytical consequence, because every network was trained on the annotated frames of its own batch alone. Annotation was confined to the first 72 h of fermentation in every batch; the 307 observations recorded after 72 h (26.8% of the 1,147 analysed observations) are therefore outside the annotated temporal domain. Data augmentation was applied online to training frames only; validation frames were never augmented. The spatial calibration marker was annotated as a label category but was not used as a training class; the pixel-to-millimetre factor was obtained by manual two-point selection on that marker in a reference frame of each acquisition series.

Table S3. Properties of the partition used for per-batch model development.

**Property**	**Value**
Number of models trained	12, one per fermentation
Pooled model trained on frames from several fermentations	None
Partition level	Individual frame, within a single fermentation
Partition method	Random permutation of that fermentation's frame list
Training fraction	round(0.8 × n) frames of the batch
Training frames, primary 11-batch inventory	166
Training frames, all 12 fermentations	177
Validation frames, primary 11-batch inventory	43
Validation frames, all 12 fermentations	46
Validation frames per fermentation	3 to 5
Held-out test subset	None defined for any batch
Grouping or stratification by batch, extract level or time window	None
Fixed random seed	Not set
Augmentation	Applied online to the training frames only
Segmentation metric computed on an unseen fermentation	None
Segmentation metric computed on same-batch validation frames	Available for 9 of 12 fermentations (Table S5)

Note: across all 12 fermentations the annotated frames were partitioned into 177 training and 46 validation frames, as reported in Table S2. Of these, 166 training and 43 validation frames are the subtotals of the 209-frame primary annotation inventory from 11 batches, and the remaining 11 training and 3 validation frames belong to the twelfth fermentation. Because every model was trained and validated on frames drawn from the same fermentation, and because consecutive

frames of one fermentation are visually highly similar, the training and validation subsets are not independent and the values in Table S5 are optimistic relative to any estimate that would be obtained on a fermentation not represented in training. Establishing generalisation to unseen fermentations requires a partition at the level of complete fermentation batches together with an independent test subset, which was not implemented for these per-fermentation models. Such a partition was implemented separately for the targeted class-imbalance experiment reported in Table S7, which trains a different, pooled model and therefore does not validate the per-fermentation models retrospectively. Extending the batch-level design to the descriptor-generating models is identified in Section 4.4 as the principal methodological priority for further work.

Table S4. Linear mixed-effects sensitivity analysis of the image-derived descriptors (70 batch-by-window cells from 12 fermentation batches).

Outcome, modelled as log(1 + outcome)	Operational time window	Initial extract level	Random intercept SD (batch)	Residual SD
Foam height	$F(5, 53.10) = 72.17$; $p < 0.001$	$F(2, 8.96) = 0.71$; $p = 0.520$	0.207	0.323
Foam-free exposed area	$F(5, 62.00) = 80.57$; $p < 0.001$	$F(2, 62.00) = 0.92$; $p = 0.404$	0.000 (singular)	1.191

Note: each outcome was aggregated within fermentation batch and operational time window and transformed as log(1 + outcome), giving 70 non-empty cells of the 12 × 6 design; two cells are empty because batch 13.03.2025 contributed no observation to the Growth window and batch 14.05.2025 none to the High Kräusen window. All 12 fermentations are included; for 07.05.2025, window assignment used the reconstructed hourly sequence validated against the image time stamps. The restriction to 11 batches reported in Table S1 applies only to the batch-level Pearson reassessment of the correlations involving fermentation time. Models included operational time window and initial extract level as fixed effects and fermentation batch as a random intercept, and were fitted by restricted maximum likelihood (MATLAB fitlme, Statistics and Machine Learning Toolbox); denominator degrees of freedom for the fixed-effect F tests were obtained by the Satterthwaite approximation. For foam-free exposed area the random-intercept variance was estimated at zero, so that model reduces to ordinary least squares and its denominator degrees of freedom equal the residual degrees of freedom of the fixed-effect design (70 − 8 = 62); this is stated explicitly because it means the exposed-area data show no detectable batch-level clustering once the time window is accounted for. This sensitivity analysis addresses clustering and temporal pseudoreplication in the 1,147 hourly observations and supersedes the observation-level correlations of Table S1 as the principal inferential evidence. The non-significant effects of initial extract level indicate that an effect was not detected with 5, 4 and 3 batches per level; they do not demonstrate the absence of an effect. Reproduction: analysis_v3.py reads the 12 Fermentatia_*.xlsx source files and writes batch_window_means.csv; mixed_model_batch_window.m then fits the two models and prints the values in this table. In accordance with the Data Availability Statement, both scripts, the aggregated input table and a README describing the data provenance and the window thresholds are not deposited publicly and are not submitted as publishable Supplementary Material, because they are subject to ongoing institutional technology-transfer and commercialisation procedures; the values they produce are reported in full in this table.

Table S5. Archived per-batch class-wise segmentation evaluation.

Fermentation	°P	Mean accuracy	Mean IoU	Foam accuracy	Foam IoU	Exposed-area accuracy	Exposed-area IoU
19.03.2025	15	0.576	0.323	0.533	0.214	0.825	0.298
26.03.2025	15	0.456	0.214	0.743	0.561	0.642	0.192
02.04.2025	18	0.450	0.213	0.681	0.330	0.527	0.139

09.04.2025	12	0.257	0.129	0.802	0.361	0.722	0.406
07.05.2025	15	0.230	0.081	0.969	0.249	0.145	0.099
14.05.2025	18	0.414	0.227	0.811	0.598	0.714	0.509
05.06.2025	12	0.438	0.226	0.835	0.739	0.920	0.272
01.07.2025	18	0.164	0.089	0.000	0.000	0.952	0.502
10.07.2025	12	0.436	0.210	0.833	0.288	0.601	0.516
Mean ± SD (n = 9)	—	0.380 ± 0.133	0.190 ± 0.077	0.690 ± 0.285	0.371 ± 0.226	0.672 ± 0.243	0.326 ± 0.164

Note: class-wise accuracy and intersection-over-union (IoU) as recorded in the archived evaluation output of the per-batch segmentation models. Mean accuracy and mean IoU are averaged over the classes each model actually used. The archived networks are not uniform in this respect: eight of the twelve were trained on all six classes (foam, foam-free exposed area, residual gas, liquid, metal, background), the model for 19.03.2025 on five (metal absent) and the models for 13.03.2025, 22.05.2025 and 22.07.2025 on four (metal and background absent). Of the nine models with archived evaluation, eight are six-class and one (19.03.2025) is five-class; the three four-class models are precisely those for which no evaluation output is archived. The class-weighting settings differ in the same way: the nine six- and five-class models carry numerical inverse-frequency class weights, whereas in the three four-class models the class-weighting field of the pixel-classification layer is set to 'none', so class weighting was not in force for them. Among the weighted models the weights are themselves uneven; in two of them (01.07.2025 and 10.07.2025) the foam class carries a weight of zero, which is consistent with 01.07.2025 being the fermentation in which the foam class was not recovered at all. These settings were read directly from the saved networks; the foam and exposed-area columns give the two classes from which the reported morphometric descriptors are derived. Each value was obtained on validation frames from the same fermentation as the training frames (3 to 5 frames; Table S3), so it is an optimistic within-batch figure and not an estimate of performance on an unseen fermentation. No evaluation output is archived for the remaining three fermentations (13.03.2025, 22.05.2025 and 22.07.2025). The low and highly variable values are consistent with each model having been trained on only 11 to 19 annotated frames. In fermentation 01.07.2025 the foam class was not recovered at all. These figures are the reason the main text reports the image-derived descriptors as relative, within-batch indicators of foam dynamics and claims no absolute measurement accuracy for them.

Table S6. Catalogue of expected failure modes in image-based foam-height estimation.

**Failure mode**	**Visual signature in the frame**	**Expected effect on the foam-height estimate**
Specular reflection on the curved glass wall	Saturated bright band, usually near the illumination axis, overlapping the foam–liquid boundary	Upper boundary detected above the true foam surface; foam height overestimated
Condensation and dried residue on the inner wall	Diffuse translucent film whose texture resembles fine foam	Foam class extended above the actual layer; overestimation that grows as fermentation proceeds
Foam adhering above the bulk interface (cling)	Isolated foam patches separated from the main layer by exposed liquid	Boundary attached to the highest patch rather than to the coherent layer; overestimation
Fragmentation of the foam boundary during collapse	Discontinuous, ragged upper contour with gaps	Peak detection on the row-integrated intensity profile becomes unstable; large errors in either direction
Partial occlusion by impeller shaft, sensors or lid ports	Grey metal regions crossing the foam layer	Foam pixels assigned to the metal class; foam height underestimated
Very thin foam layer in the Lag and Final windows	Foam occupying only a few pixel rows	Class imbalance dominates and the foam class may not be recovered at all, as in one fermentation (Table S5)

Illumination drift over a long fermentation	Gradual change in overall frame brightness between successive acquisitions	Systematic drift of the detected boundary; batch-specific bias rather than random error

Note: this catalogue is qualitative. The frequency of each failure mode was not quantified in this study, and the table is offered as a structured statement of the error sources expected for the present acquisition geometry rather than as measured error decomposition. Modes 1 to 3 tend to bias the estimate upwards and mode 5 downwards, while modes 4, 6 and 7 may introduce direction-dependent error or increased variability. Together they are the reason the frame-level foam-height values are interpreted as relative indicators; quantifying them would require a set of frames annotated specifically for these conditions, which the present annotation inventory (Table S2) does not contain.

Table S7. Targeted class-imbalance experiment on a fixed batch-level partition.

**Configuration**	**Architecture**	**Loss**	**Mean IoU**	**Foam IoU**	**Foam Dice**	**Foam recall**	**Foam precision**	**Δ foam IoU per frame**	**p**
Baseline	encoder–decoder	weighted cross-entropy	0.205	0.460	0.630	0.540	0.757	—	—
Tversky	encoder–decoder	Tversky (0.7/0.3)	0.178	0.498	0.665	0.661	0.668	+0.043	0.005
Attention	attention U-Net	weighted cross-entropy	0.309	0.448	0.619	0.548	0.711	+0.001	0.870
Attention + Tversky	attention U-Net	Tversky (0.7/0.3)	0.302	0.524	0.688	0.639	0.744	+0.054	<0.001

Sparse-foam frames (n = 8, below 5% foam pixels)

**Configuration**	**Foam IoU**	**Foam Dice**	**Foam recall**	**Frames with no foam recovered**
Baseline	0.043	0.075	0.074	1 / 8
Tversky	0.083	0.147	0.318	0 / 8
Attention	0.078	0.131	0.215	1 / 8
Attention + Tversky	0.056	0.093	0.098	4 / 8

Foam IoU in each withheld fermentation, evaluated separately

**Configuration**	**09.04.2025 (12°P)**	**26.03.2025 (15°P)**	**02.04.2025 (18°P)**
Baseline	0.456	0.520	0.200
Tversky	0.474	0.533	0.286
Attention	0.429	0.487	0.248
Attention + Tversky	0.510	0.557	0.264

Note: four configurations were trained on a fixed partition defined at the level of complete fermentations (seven training, two validation and three withheld test fermentations; 223 annotated frames in total, of which 142 training, 28 validation and 53 test). Test fermentations were 09.04.2025 (12°P), 26.03.2025 (15°P) and 02.04.2025 (18°P); no frame from a test fermentation contributed to training or validation. Everything other than the architecture and the loss function was held identical across the four runs, including the random seed, the augmentation pipeline, the optimiser and its schedule, the mini-batch size, regularisation, input resolution, early-stopping criterion and model selection (Section 2.5.3). Metrics are computed on the 53 test frames, of which 52 contain foam in the ground truth; unannotated pixels (21.7% of all pixels) are excluded. Δ foam IoU per frame is the mean paired difference against the baseline over the 52 foam-containing frames, with p from the

Wilcoxon signed-rank test; 95% percentile bootstrap confidence intervals were 0.016 to 0.069 (Tversky), −0.020 to 0.020 (Attention) and 0.040 to 0.067 (Attention + Tversky), with effect sizes r = 0.39, 0.02 and 0.73 respectively. Because all 52 frames derive from only three independent fermentations, this is a paired frame-level analysis conditional on those three batches and not inference over independent experimental units; it is reported as technical support for the comparison, and the per-fermentation values given below allow the direction of the effect to be checked in each withheld batch separately. The attention-gated configuration adds skip connections and attention gates together, so its effect cannot be attributed to the gating mechanism alone. These results rest on a single partition and a single random seed, and the sparse-foam comparison rests on eight frames, so the magnitudes are indicative rather than stable estimates of effect. The experiment trains one pooled model and therefore is not directly comparable with the archived per-batch evaluation of Table S5, which it does not supersede. In accordance with the Data Availability Statement, the training and evaluation code, the annotated frames and the trained networks are not deposited publicly and are not submitted as publishable Supplementary Material, because they are subject to ongoing institutional technology-transfer and commercialisation procedures; the complete evaluation metrics obtained from them on the withheld fermentations are reported in this table and in Figure S1. They can be made available confidentially to the Editorial Office on request.

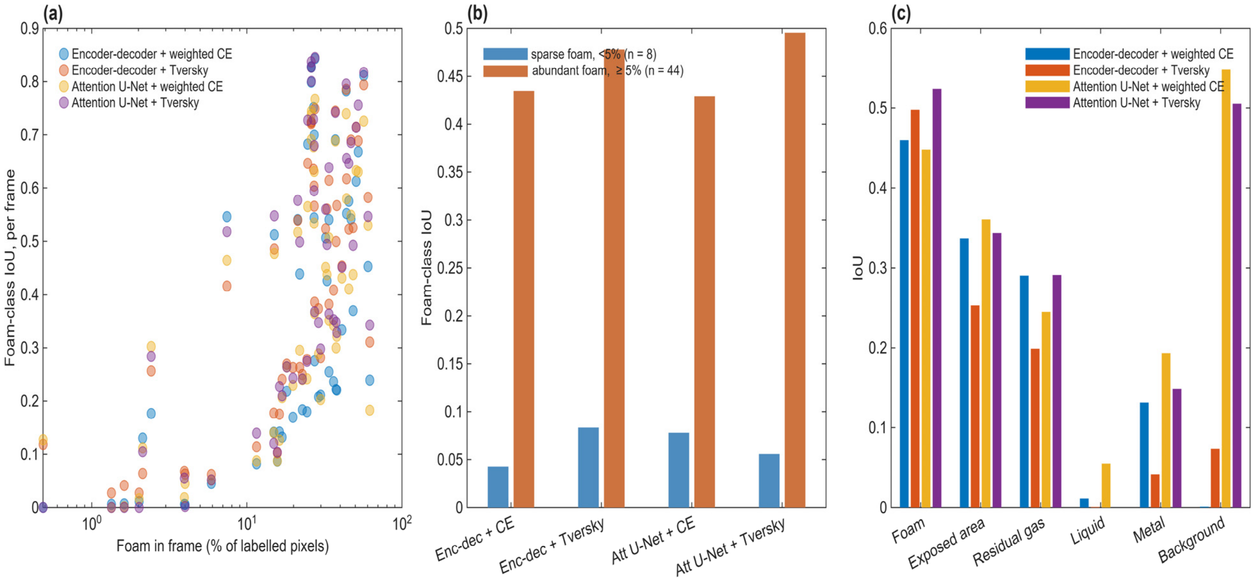

Figure S1. Targeted class-imbalance experiment on the three withheld fermentations. (a) Per-frame foam intersection-over-union as a function of the proportion of foam pixels in the frame, on a logarithmic axis; performance degrades sharply below approximately 5% foam for all four configurations. (b) Foam intersection-over-union separated into sparse (<5%) and abundant (≥5%) frames; the Tversky loss on the encoder–decoder gives the largest gain in the sparse regime. (c) Per-class intersection-over-union; the attention-gated configuration improves the background class markedly, whereas the Tversky loss improves the foam class at the expense of the abundant classes. Because skip connections and attention gates were introduced together, the multi-class gain cannot be attributed to the gating mechanism alone. CE = class-weighted cross-entropy; Enc-dec = the archived encoder–decoder network; Att U-Net = attention U-Net.
